# Compartment-specific adaptive responses and dysregulation under NQO1 deficiency in diabetic kidney disease: A transcriptomic GSEA-based investigation

**DOI:** 10.1371/journal.pone.0331582

**Published:** 2025-09-08

**Authors:** Yongwoo Lee, Sang-Hee Lee, Eunyoung Moon, Hyerim Park, Janghyun Jo, Jung Hwan Hwang, Dae Eun Choi

**Affiliations:** 1 Department of Nephrology, Chungnam National University, Daejeon, Republic of Korea; 2 Center for Bio-imaging and Translational Research, Korea Basic Science Institute (KBSI), Cheongju, Republic of Korea; 3 Department of Medical Science, Chungnam National University School of Medicine, Daejeon, Republic of Korea; 4 Laboratory Animal Resource Center, Korea Research Institute of Bioscience and Biotechnology (KRIBB), Daejeon, Republic of Korea; King Faisal University College of Veterinary Medicine and Animal Resources, SAUDI ARABIA

## Abstract

Diabetic kidney disease (DKD) involves oxidative stress–driven damage to glomeruli (Gloms) and proximal convoluted tubules (PCT). NAD(P)H: quinone oxidoreductase 1 (NQO1) regulates redox balance, but its compartment-specific role remains unclear. Streptozotocin (STZ)-induced hyperglycemia increased albuminuria and foot process effacement, with NQO1 KO (NKO) mice exhibiting greater podocyte injury than WT, indicating exacerbated glomerular damage. To investigate the underlying mechanisms, we conducted compartment-specific transcriptomic Gene Set Enrichment Analysis (GSEA) in Gloms and PCT. In Gloms, ribosome biogenesis and immune pathways were upregulated in WT-STZ compared to WT but suppressed in NKO-STZ compared to STZ, indicating impaired protein synthesis and immune regulation in NQO1 deficiency. In PCT, ribosome activity, oxidative phosphorylation, glutathione metabolism, and cytoskeletal pathways were elevated in WT-STZ compared to WT but suppressed in NKO-STZ compared to WT-STZ. However, ribosome activity was relatively less affected than in Gloms. Additionally, adherens junction activation was more pronounced in WT-STZ Gloms than in NKO mice Gloms, suggesting a compensatory mechanism to maintain podocyte foot process integrity. This response involved key cytoskeletal genes, including Actg1, Ctnna1, Tjp1, Rhoa, and Iqgap1. These findings highlight compartment-specific adaptive responses to STZ-induced hyperglycemia and underscore NQO1’s role in regulating these adaptations. Our results suggest that enhancing NQO1 activity may restore redox balance and preserve nephron integrity, supporting its potential as a therapeutic target for DKD. Furthermore, the observed compartment-specific responses highlight the need for precision redox therapies tailored to glomerular and tubular vulnerabilities.

## Introduction

Diabetic kidney disease (DKD) remains a leading cause of end-stage renal disease worldwide, posing a significant clinical and economic burden [1,2]. Under hyperglycemic conditions, excessive production of reactive oxygen species (ROS) disrupts endogenous antioxidant defenses, leading to oxidative stress, inflammation, and progressive remodeling of renal structures [3,4]. While the glomerulus has traditionally been the primary focus—due to its crucial role as the filtration barrier—growing evidence highlights that tubulointerstitial injury is equally critical in DKD pathogenesis and progression [5–7]. In particular, the proximal convoluted tubule (PCT) plays a pivotal role in maintaining kidney function by facilitating essential reabsorptive and metabolic processes. Damage to the tubular compartments can accelerate renal functional decline in diabetes.

Among the key modulators of DKD pathophysiology, NAD(P)H: quinone oxidoreductase 1 (NQO1) has emerged as a crucial factor in ROS clearance and cellular redox homeostasis [8–10]. By catalyzing the two-electron reduction of quinones to hydroquinones, NQO1 helps limit superoxide generation and stabilizes intracellular NAD + /NADH levels. This enzymatic function is transcriptionally regulated by the nuclear factor erythroid 2–related factor 2 (Nrf2) pathway, a master regulator of antioxidant defense.[11] Hyperglycemia elevates intracellular reactive oxygen species (ROS) through multiple mechanisms, including the polyol pathway, glucose autoxidation, NADPH oxidase activation, and AGE formation [12]. This increased oxidative burden activates Nrf2 signaling by promoting its dissociation from Keap1 and nuclear translocation, where it induces antioxidant response element (ARE)-driven genes including NQO1 [13,14]. Thus, NQO1 acts as a downstream effector of the Nrf2-mediated defense system, contributing to redox adaptation under diabetic conditions [13].

The maintenance of NAD+ homeostasis, in turn, regulates NAD + -dependent enzymes such as sirtuin-1, which modulates oxidative stress, apoptosis, and inflammation in diabetic kidneys [15–18]. Despite substantial evidence of its role in other oxidative stress–related disorders, NQO1’s compartment-specific contribution in the glomerulus and PCT under hyperglycemic conditions remains poorly understood.

The glomeruli (Gloms) consist of specialized cells, particularly podocytes, that form the filtration barrier and are highly susceptible to mechanical and oxidative insults. Podocyte injury is closely linked to proteinuria and the progression of glomerular disease in DKD [6,7]. Meanwhile, PCT cells, which are persistently exposed to high-glucose environments, may exhibit increased protein synthesis in response to STZ-induced injury, serving as both a partial repair mechanism and a hypertrophic adaptation [19,20]. Previous studies suggest that enhanced ribosomal activity in PCT initially supports structural and metabolic demands under hyperglycemia; however, prolonged activation may drive progressive hypertrophy and hyperfiltration, ultimately accelerating DKD [20]. Thus, while the reabsorptive function of PCT is essential, dysregulation of these adaptive or maladaptive mechanisms may lead to tubulointerstitial fibrosis and chronic kidney dysfunction [5,6].

Given its central role in redox detoxification and NAD ⁺ /NADH balance, NQO1 has emerged as a potential therapeutic target in metabolic diseases including DKD. As a downstream effector of the Nrf2 pathway—currently being targeted in clinical trials with agents such as bardoxolone methyl—NQO1’s modulation may offer compartment-specific protection against hyperglycemia-induced injury [21–23]. However, the extent to which NQO1 deficiency exacerbates compartment-specific injury remains unclear [10,24]. Based on the distinct structural and metabolic characteristics of the glomerulus and proximal tubule, we hypothesized that NQO1 plays a protective role against hyperglycemia-induced oxidative stress in a compartment-specific manner. In the glomerulus, we expected NQO1 deficiency to aggravate podocyte injury and cytoskeletal disruption, while in the proximal tubule, we anticipated impaired biosynthetic and immune responses due to high metabolic demand. This study aimed to test these hypotheses using spatially resolved transcriptomic analysis in STZ-induced diabetic WT and NQO1-knockout(NKO) mice.

By characterizing compartment-specific transcriptomic responses to NQO1 deficiency, we provide molecular insights supporting the development of precision redox therapies tailored to vulnerable nephron segments.

## Methods

### Study design and experimental approach

This study investigated the compartment-specific impact of NQO1 deficiency in diabetic kidney disease (DKD) using a streptozotocin (STZ)-induced diabetes model in wild-type (WT) and NQO1-knockout (NKO) mice. The experimental design included non-diabetic (WT, NKO) and diabetic (WT-STZ, NKO-STZ) groups to examine how NQO1 deficiency influences molecular and cellular responses under hyperglycemia. Urinary albumin-to-creatinine ratio (ACR) was measured to assess kidney function, and kidney tissues were collected to evaluate foot process effacement and glomerular basement membrane (GBM) thickness. Subsequently, transcriptomic analysis was performed on glomeruli (Gloms) and proximal convoluted tubules (PCT) to examine gene expression profiles and pathway alterations across experimental conditions and renal compartments.

### Animal models and ethical approval

Male WT and NQO1 knockout (NKO) mice with a C57BL/6N genetic background were used in this study. All animals were maintained under specific pathogen-free conditions at a stable temperature of 20°C. Littermates of NQO1 KO mice served as WT controls. All experimental procedures involving animals were conducted in accordance with standard guidelines for the care and use of laboratory animals and were approved by the Institutional Animal Care and Use Committee of Chungnam National University (IRB NO.202112A-CNU-201). Diabetic nephropathy was induced by injection of STZ in freshly prepared 0.1 M citrate buffer (pH 4.5). Eight-week-old, age-matched WT and NKO male mice underwent intraperitoneal injections of STZ at a dose of 50 mg/kg body weight following a 4-hour fasting period, administered once daily for five consecutive days. Control mice received an equivalent volume of citrate buffer. Blood glucose levels were monitored using an Accu-Chek glucometer (Roche). Kidney tissue collection was performed under anesthesia using a ketamine/xylazine mixture eight weeks after the initial STZ injection. Euthanasia was carried out using CO₂ gas in a designated euthanasia chamber.

### Albumin-to-creatinine ratio measurement

Urinary albumin excretion was assessed using the Albumin (Mouse) ELISA Kit (ALPCO, NH, USA) following the manufacturer’s guidelines. Urine creatinine levels were determined with the Toshiba 200FR chemistry autoanalyzer (Toshiba Medical Systems Co., Tokyo, Japan). The albumin-to-creatinine ratio (μg/L to mg/L) was then calculated.

### Transmission electron microscopy

Transmission electron microscopy was performed as previously described. Briefly, tissues were fixed with 1% glutaraldehyde in phosphate-buffered saline (PBS) for 2 hours at 4°C and then embedded in EMbed-812 resin (Electron Microscopy Sciences, Hatfield, PA, USA). The samples were examined using a KBSI Bio-High Voltage EM system (JEM-1400 Plus at 120 kV and JEM-1000BEF at 1000 kV: JEOL Ltd., Tokyo, Japan). Foot process effacement was assessed by counting the number of filtration slit membranes per 100 μm of the glomerular basement membrane.

### Region of interest (ROI) analysis

Kidney samples were fixed in 10% neutral-buffered formalin, embedded in paraffin, and sectioned at 4 μm thickness for ROI analysis. Tissue morphology was verified to ensure that representative areas were sampled (see S1 Fig).

- Morphology Markers: ROIs were selected based on immunostaining for markers such as Pan-cytokeratin (PanCK), CD10, and CD31, along with nuclear staining using Syto83. These staining patterns enabled the manual delineation of geometric ROIs corresponding to glomerular or proximal convoluted tubular structures.
- Geometric ROI Strategy: ROIs were defined in accordance with tissue structure and morphological features, capturing areas that reflect either injury-related changes or standard histology. This approach ensured a balanced selection of regions for transcriptomic profiling.

### Assay quality control and sensitivity

Sequencing data quality and saturation were examined to ensure adequate sensitivity and reliability. To address differences in cellular density and ROI size, data were normalized to the upper quartile (Q3) expression level. A total of 19,962 genes were initially profiled, among which 15,261 genes were detected in at least 10% of the areas of interest (AOIs), and 9,274 were expressed in 50% of AOIs. This distribution validated the robustness of the gene expression analysis and provided sufficient coverage for subsequent statistical and functional interpretation.

### Differentially expressed gene (DEG) analysis

A two-step statistical filtering process identified differentially expressed genes (DEGs). First, genes with *P* < 0.05 were selected; among these, genes meeting a false discovery rate (FDR) cutoff < 0.25 were prioritized. Genes were then ranked in ascending order of FDR for further analysis. Volcano plots visualized upregulated and downregulated genes under each experimental condition, with fold change (FC) indicating the magnitude of expression differences. Genes displaying both statistical significance and strong biological relevance were chosen for in-depth investigation.

### Gene set enrichment analysis (GSEA)

All genes with *P* < 0.05 from the volcano plot analysis were sorted by descending fold change and included in a ranked gene list for GSEA. Analyses were conducted using the clusterProfiler package (v4.14.4) with KEGG gene sets from msigdbr (category = “C2”, subcategory = “CP:KEGG”), covering a wide array of metabolic and signaling pathways curated for *Mus musculus*.

GSEA calculates a Normalized Enrichment Score (NES), which quantifies the extent to which a pathway is enriched in a ranked gene list compared to a randomly distributed set. A positive NES indicates pathway activation, whereas a negative NES suggests pathway suppression relative to the reference group. Adjusted p-values and NES values were used to determine pathway significance.

Pathways with q < 0.25 were prioritized. Pathways with q < 0.25 were prioritized. For pathways exceeding this threshold but still considered potentially relevant, we relaxed the cutoff to q < 0.50 to avoid missing biologically important signals.

### Data analysis and visualization

All data processing and statistical analyses were performed in **R** (v4.4.1) using RStudio. Preliminary filtering and organization of DEGs, as well as pathway analyses, were handled with **dplyr**, **tidyr**, and **clusterProfiler**. Final visualization—including volcano and ridge plots—was accomplished via **ggplot2** (v3.5.1) and **enrichplot** (v1.26.6), enabling clear depiction of enriched pathways and gene expression trends under each experimental setting.

## Results

### More severe glomerular injury in NKO mice under STZ-induced hyperglycemia

**Fig 1:**
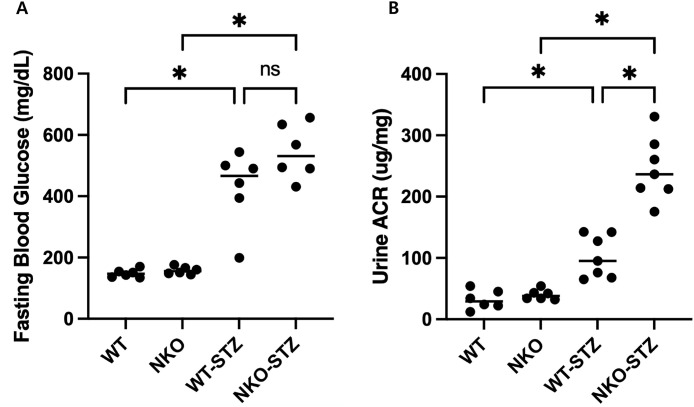
Blood glucose and albuminuria in WT and NKO mice. (A) Fasting blood glucose levels at week 8 in WT, NKO, WT-STZ, and NKO-STZ mice. STZ administration significantly increased blood glucose levels in both WT-STZ and NKO-STZ groups compared to non-STZ controls (*P < 0.05). No significant difference was observed between WT-STZ and NKO-STZ (ns). (B) Urinary albumin-to-creatinine ratio (ACR) in the same groups. STZ-treated groups exhibited a significant increase in albuminuria, with NKO-STZ showing a markedly higher ACR than WT-STZ (*P < 0.05), indicating exacerbated diabetic kidney injury under NQO1 deficiency. *Symbols indicate statistical significance (*P < 0.05*) or non-significance (ns) between groups. Data represent means ± SEM (n = 6–7 per group).

Urinary albumin-to-creatinine ratios (ACR) were significantly elevated in WT-STZ, with NKO–STZ exhibiting the highest ACR (*P* < 0.05, Fig 1B), indicating greater susceptibility to diabetic kidney injury under NQO1 deficiency.

**Fig 2:**
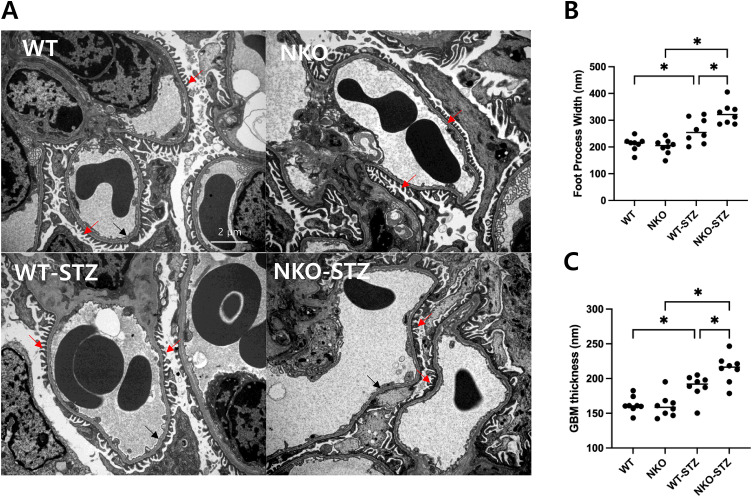
Electron microscopy reveals exacerbated podocyte injury in NKO mice under STZ treatment. (A) Representative transmission electron micrographs of glomeruli (Gloms) from WT, NKO, WT–STZ, and NKO–STZ mice. Red arrows indicate podocyte foot processes, while black arrows mark the glomerular basement membrane (GBM). Scale bar, 2 μm. (B) Quantitative assessment of foot process effacement reveals significant effacement in NKO–STZ compared to WT–STZ (*P < 0.05), indicating greater podocyte injury under NQO1 deficiency. (C) Glomerular basement membrane (GBM) thickness was increased in STZ-treated groups compared to non-STZ controls. And the difference between WT–STZ and NKO–STZ also reach statistical significance (*P < 0.05). Each dot represents an individual glomerulus from one mouse, highlighting the ultrastructural impact of NQO1 deficiency under hyperglycemic conditions.

In electron microscopy, Podocyte foot process effacement was significantly increased in NKO–STZ compared to WT–STZ (*P* < 0.05, Fig 2B), reflecting extensive effacement and podocyte structural damage. Glomerular basement membrane (GBM) thickness was increased in STZ-treated groups compared to non-STZ controls. And the difference between WT–STZ and NKO–STZ also reach statistical significance (*P* < 0.05, Fig 2C). Together, these results suggest that NQO1 deficiency exacerbates hyperglycemia-induced glomerular injury, as reflected by worsened albuminuria and structural abnormalities.

To further investigate the molecular basis of these compartment-specific injuries, we performed differential gene expression (DEG) and Gene Set Enrichment Analysis (GSEA), separately analyzing Gloms and PCT. This approach allowed us to delineate distinct adaptive and maladaptive responses within each renal segment under STZ-induced diabetic stress.

### Differential gene expression analysis

**Fig 3:**
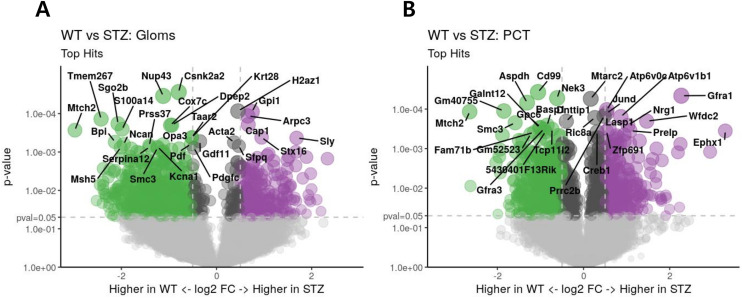
Volcano plots of differential gene expression. (A) Differentially expressed genes in Gloms. (B) Differentially expressed genes in proximal convoluted tubules (PCT) from WT and WT-STZ mice. Significance thresholds were set at *P* < 0.05 and FDR < 0.50 for Gloms and *P* < 0.05 and FDR < 0.25 for PCT. The x-axis represents the log₂ fold change based on log₂-transformed Q3-normalized counts, while the y-axis shows the corresponding P-values on a –log₁₀ scale.

To elucidate gene-level changes in Gloms under diabetic stress, we compared WT-STZ versus WT samples using a threshold of *P* < 0.05 and FDR < 0.50. (An FDR cutoff of 0.50 was used as an exception because no genes met the more stringent FDR < 0.25 criterion.) Under these parameters, we identified 17 genes that were significantly upregulated (fold change > 1) and 120 genes that were significantly downregulated (fold change < 1) in the STZ group..(see Fig 3, which shows volcano plots of the differentially expressed genes in Gloms and PCT) Notable upregulated genes—*Sly*, *Zmat3*, *Fam168a*, and *Cd74*—suggest heightened protein synthesis or stress-response pathways, whereas the downregulation of *Csnk2a2* and *Nup43* points to potential disruptions in mitochondrial function or cell-cycle regulation. Collectively, these results imply that while STZ-induced injury triggers partial adaptive responses (e.g., increased protein synthesis and cytoskeletal reorganization), it also exacerbates metabolic and oxidative stress in the glomerular compartment.

A similar analysis of PCT revealed 14 upregulated and 112 downregulated genes in STZ-treated mice. Here, the induction of *Gfra1* and *Nrg1* may reflect adaptive metabolic or growth-factor signaling in response to hyperglycemia, whereas decreased expression of *Aspdh* and *Mtch2* suggests compromised mitochondrial function and heightened oxidative challenges. Given the relatively high false discovery rates for several individual genes, we employed a pathway-oriented approach—specifically Gene Set Enrichment Analysis (GSEA)—to derive broader mechanistic insights and refine our interpretation of these compartment-specific gene expression profiles.

### WT vs. WT-STZ comparison

**Fig 4:**
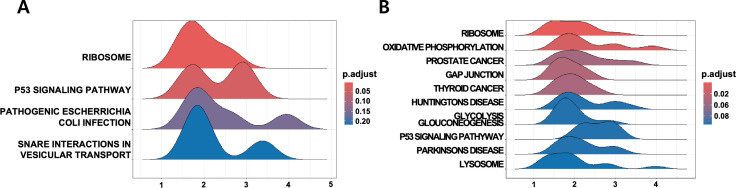
Ridge plots of the top 10 enriched KEGG pathways (q < 0.25) in WT vs. WT-STZ comparisons. (A) Gloms and (B) PCT. The ridge plots display the distribution of normalized enrichment scores (NES) for the top 10 KEGG pathways enriched in each renal compartment. The x-axis represents NES values, while the color gradient reflects the adjusted p-value (p.adjust). These visualizations illustrate the extent and variability of pathway activation in response to STZ-induced diabetic conditions in Gloms and PCT.

Gene Set Enrichment Analysis (GSEA) revealed that in Gloms, the Ribosome pathway was significantly elevated (NES = 2.92, *P*-adjust = 3.80 × 10 ⁻ ⁴, q = 3.72 × 10 ⁻ ⁴), reflecting intensified protein synthesis under hyperglycemic conditions..(See Fig 4 for a visual representation of the top 10 representative pathways, with corresponding enrichment details summarized in Table 1 (Gloms) and Table 2 (PCT)) Notably, previous work in high-glucose-treated glomerular epithelial cells showed that augmented ribosome biogenesis (via UBF-mediated rRNA transcription) can facilitate increased protein synthesis and contribute to hypertrophy in diabetic settings [20]. Concomitant activation of p53 signaling pointed to increased DNA damage responses and pro-apoptotic signals, while immune/inflammatory pathways (e.g., *Pathogenic E. coli* infection, NES = 2.00) indicated heightened immune reactions.

**Table 1:** Gene set enrichment analysis (GSEA) of gloms WT vs. WT-STZ (q-value < 0.25).

Pathway	Set Size	NES	P-value	P-adjust	Q-value	Core Gene Names
KEGG_RIBOSOME	14	2.92	2.64E-06	3.80E-04	3.72E-04	Rps9/Rpl34/Rpl10a/Rps3/Rpl14/Rpl27/Rpl37/Rpl28/Rps23/Rps17/Rps3a1/Rpl13
KEGG_P53_SIGNALING_PATHWAY	7	2.29	2.63E-04	1.89E-02	1.85E-02	Zmat3/Sesn3/Cdkn1a/Steap3/Gtse1/Ccng1
KEGG_PATHOGENIC_ESCHERICHIA_COLI_INFECTION	7	2	3.21E-03	1.54E-01	1.51E-01	Arpc3/Actg1/Arpc1b/Arpc4/Cttn
KEGG_SNARE_INTERACTIONS_IN_VESICULAR_TRANSPORT	5	1.91	5.20E-03	1.87E-01	1.84E-01	Stx16/Vamp2/Stx3/Snap29

Significantly enriched KEGG pathways with normalized enrichment score (NES), p-value, p-adjust, q-value, and representative genes.

**Abbreviations:** NES, normalized enrichment score; p-adjust, adjusted p-value; KEGG, Kyoto Encyclopedia of Genes and Genomes.

**Table 2:** Gene set enrichment analysis (GSEA) of proximal convoluted tubules (PCT) from WT vs. WT-STZ (q-value < 0.25).

Pathway	Set Size	NES	P-value	P-adjust	Q-value	Core Gene Names
KEGG_RIBOSOME	37	3.85	1.00E-10	1.66E-08	1.43E-08	Rpl10a/Rpl24/Rps7/Rpl27a/Rpl27/Rpl28/Rpl4/Rpl13a/Rps9/Rpl37/Rps2/Rpl7a/Rpl14/Rpl38/Rps3/Rpl18a/Rps4x/Rps11/Rpl22/Rpl31/Rps12/Rplp0/Rps10/Rps27l/Rpl34/Rpl19/Rpl17/Rps17/Rps13/Rpl13/Rpl18/Rps21/Rpl26/Rps3a1/Rps23/Rplp1
KEGG_OXIDATIVE_PHOSPHORYLATION	22	2.46	1.77E-04	1.47E-02	1.26E-02	Atp6v0c/Atp6v1b1/Cox6b1/Atp5c1/Ndufa8/Atp5g2/Ndufb1/Atp5o/Atp6v1e1/Atp5a1/Cox6b2/Ndufb6/Atp5j2/Atp5d/Uqcr11
KEGG_PROSTATE_CANCER	11	2.19	1.28E-03	3.65E-02	3.15E-02	Creb1/Cdkn1a/Ctnnb1/Mdm2/Ccnd1/Mapk3/Gstp1/Mapk1/Egf
KEGG_GAP_JUNCTION	15	2.17	1.11E-03	3.65E-02	3.15E-02	Tuba1b/Gucy1a1/Gucy1a2/Mapk3/Prkaca/Gnas/Tuba4a/Adcy6/Mapk1/Gnai2/Egf
KEGG_THYROID_CANCER	6	2.15	6.76E-04	3.65E-02	3.15E-02	Ctnnb1/Ccnd1/Mapk3/Rxra/Tpm3/Mapk1
KEGG_HUNTINGTONS_DISEASE	20	2.12	1.36E-03	3.65E-02	3.15E-02	Creb1/Cox6b1/Atp5c1/Ndufa8/Atp5g2/Ndufb1/Atp5o/Atp5a1/Cox6b2/Gpx1/Ndufb6/Atp5d/Uqcr11
KEGG_GLYCOLYSIS_GLUCONEOGENESIS	13	2.05	1.54E-03	3.65E-02	3.15E-02	Gpi1/Tpi1/Akr1a1/Aldh7a1/Aldh2/Eno1/Pgk1/Pfkp/Ldhb
KEGG_LYSOSOME	16	1.97	2.60E-03	5.39E-02	4.65E-02	Atp6v0c/Laptm4b/Sumf1/Ids/Fuca1/Ctsd/Igf2r/Psap/Cd164/Clta/Ctsa/Gns/Atp6ap1
KEGG_P53_SIGNALING_PATHWAY	8	2.05	3.79E-03	5.91E-02	5.09E-02	Ccng1/Cdkn1a/Zmat3/Mdm2/Ccnd1
KEGG_PARKINSONS_DISEASE	21	1.99	4.27E-03	5.91E-02	5.09E-02	Cox6b1/Atp5c1/Ndufa8/Uchl1/Atp5g2/Ndufb1/Atp5o/Atp5a1/Cox6b2/Ndufb6/Ube2l3/Atp5d/Uqcr11
KEGG_FOCAL_ADHESION	24	1.96	3.87E-03	5.91E-02	5.09E-02	Ctnnb1/Flnc/Ccnd1/Mapk3/Col4a2/Myl12a/Parva/Xiap/Col3a1/Zyx/Pak1/Mapk1/Egf/Col4a1/Actn4/Flna/Actb/Reln
KEGG_ALZHEIMERS_DISEASE	24	1.95	4.38E-03	5.91E-02	5.09E-02	Cox6b1/Atp5c1/Ndufa8/Atp5g2/Ndufb1/Atp5o/App/Atp5a1/Mapk3/Cox6b2/Ndufb6/Atp5d/Uqcr11/Mapk1/Lrp1/Calm1/Ndufs3
KEGG_FRUCTOSE_AND_MANNOSE_METABOLISM	6	1.92	4.96E-03	5.91E-02	5.09E-02	Tpi1/Sord/Mpi/Pfkp/Gfus/Hk1
KEGG_UBIQUITIN_MEDIATED_PROTEOLYSIS	16	1.87	4.98E-03	5.91E-02	5.09E-02	Cblc/Anapc11/Ube2s/Mdm2/Sae1/Xiap/Ube2l3/Ube2i/Cdc16/Nedd4
KEGG_CHRONIC_MYELOID_LEUKEMIA	8	1.97	5.76E-03	5.98E-02	5.15E-02	Cblc/Cdkn1a/Mdm2/Ccnd1/Mapk3
KEGG_MELANOGENESIS	13	1.88	5.61E-03	5.98E-02	5.15E-02	Creb1/Ctnnb1/Mapk3/Prkaca/Gnas/Fzd7/Adcy6/Mapk1/Gnai2
KEGG_VASOPRESSIN_REGULATED_WATER_REABSORPTION	8	1.97	6.22E-03	5.99E-02	5.16E-02	Creb1/Prkaca/Gnas/Rab5c/Vamp2/Adcy6/Rab5a
KEGG_ADHERENS_JUNCTION	9	1.92	6.57E-03	5.99E-02	5.16E-02	Ctnnb1/Nectin2/Mapk3/Acp1/Mapk1/Actn4/Actb/Farp2/Nectin3
KEGG_SNARE_INTERACTIONS_IN_VESICULAR_TRANSPORT	6	1.87	6.85E-03	5.99E-02	5.16E-02	Gosr2/Stx1a/Vamp2/Stx17/Vamp7/Vamp5
KEGG_NON_SMALL_CELL_LUNG_CANCER	8	1.92	7.62E-03	6.32E-02	5.45E-02	Ccnd1/Mapk3/Rxra/Foxo3/Mapk1/Egf/Rarb
KEGG_ENDOMETRIAL_CANCER	9	1.89	8.11E-03	6.41E-02	5.53E-02	Ctnnb1/Ccnd1/Axin1/Mapk3/Foxo3/Mapk1/Egf
KEGG_GLUTATHIONE_METABOLISM	9	1.87	9.50E-03	7.17E-02	6.18E-02	Pgd/Gsta3/Gstm2/Mgst1/Gstp1/Gpx1
KEGG_BLADDER_CANCER	7	1.87	1.25E-02	8.30E-02	7.16E-02	Cdkn1a/Mdm2/Ccnd1/Mapk3/Mapk1/Egf
KEGG_VIBRIO_CHOLERAE_INFECTION	8	1.84	1.24E-02	8.30E-02	7.16E-02	Atp6v0c/Atp6v1b1/Atp6v1e1/Prkaca/Gnas
KEGG_CELL_CYCLE	13	1.76	1.20E-02	8.30E-02	7.16E-02	Cdkn1a/Anapc11/Ywhah/Mdm2/Ccnd1/Ywhae/Bub3
KEGG_MELANOMA	8	1.78	1.64E-02	1.05E-01	9.03E-02	Cdkn1a/Mdm2/Ccnd1/Mapk3/Mapk1/Egf
KEGG_PENTOSE_PHOSPHATE_PATHWAY	5	1.69	1.82E-02	1.12E-01	9.67E-02	Gpi1/Pgd/Pfkp/Tkt
KEGG_PATHOGENIC_ESCHERICHIA_COLI_INFECTION	8	1.7	2.55E-02	1.46E-01	1.26E-01	Ctnnb1/Tuba1b/Tuba4a/Ncl/Tubb4b/Arpc2/Actb
KEGG_CITRATE_CYCLE_TCA_CYCLE	5	1.63	2.51E-02	1.46E-01	1.26E-01	Idh3g/Mdh2/Mdh1/Suclg1/Pdha2
KEGG_PATHWAYS_IN_CANCER	31	1.67	2.74E-02	1.52E-01	1.31E-01	Cblc/Cdkn1a/Ctnnb1/Mdm2/Ccnd1/Axin1/Mapk3/Rxra/Col4a2/Gstp1/Xiap/Fzd7/Tpm3/Mapk1/Egf/Col4a1/Rarb
KEGG_O_GLYCAN_BIOSYNTHESIS	4	−1.54	5.22E-02	2.59E-01	2.23E-01	Galnt15/Gcnt1/Galnt12
KEGG_ASCORBATE_AND_ALDARATE_METABOLISM	2	1.46	5.30E-02	2.59E-01	2.23E-01	Aldh7a1/Aldh2
KEGG_BETA_ALANINE_METABOLISM	2	1.46	5.30E-02	2.59E-01	2.23E-01	Aldh7a1/Aldh2
KEGG_LIMONENE_AND_PINENE_DEGRADATION	2	1.46	5.30E-02	2.59E-01	2.23E-01	Aldh7a1/Aldh2
KEGG_LONG_TERM_DEPRESSION	10	1.58	5.83E-02	2.77E-01	2.39E-01	Gucy1a1/Gucy1a2/Mapk3/Gnas/Ppp2ca/Mapk1/Gnai2

Significantly enriched KEGG pathways in PCT under STZ-induced diabetic conditions, listed with normalized enrichment score (NES), p-value, p-adjust, q-value, and representative genes.

**Abbreviations:** NES, normalized enrichment score; p-adjust, adjusted p-value; KEGG, Kyoto Encyclopedia of Genes and Genomes.

In PCT, the Ribosome pathway showed even stronger upregulation (NES = 3.85, *P*-adjust = 1.66 × 10 ⁻ ⁸, q = 1.43 × 10 ⁻ ⁸), coupled with oxidative phosphorylation, glutathione metabolism, and glycolysis/gluconeogenesis—signifying a robust adaptive response to oxidative stress. Gap junction and Lysosome pathways were also enriched, implying increased intercellular communication and the clearance of damaged proteins.

### Tissue-specific response

**Fig 5:**
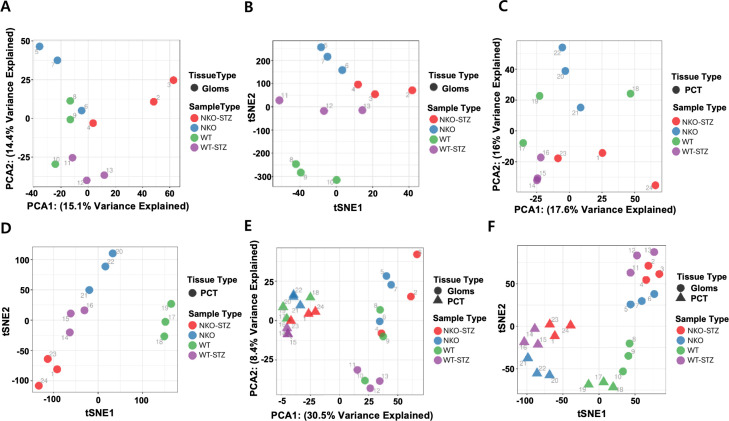
Dimensional reduction plots of Gloms and PCT. (A) Principal component analysis (PCA) of Gloms. (B) t-distributed stochastic neighbor embedding (t-SNE) of Gloms. (C) PCA of PCT. (D) t-SNE of PCT. (E) PCA of combined Gloms and PCT. (F) t-SNE of combined Gloms and PCT, comparing WT, NKO, WT-STZ, and NKO-STZ groups. PCA and t-SNE were applied to visualize large-scale variability in gene expression across these groups. Both PCA and t-SNE plots show clear separation between conditions in Gloms and PCT, with distinct clustering in Gloms samples indicating differential molecular responses to diabetic injury.

Compared to PCT, Gloms exhibited more pronounced immune/inflammatory pathways, underscoring their heightened sensitivity to hyperglycemic stress. Meanwhile, PCT demonstrated stronger activation of antioxidant and metabolic pathways, emphasizing its critical role in reabsorption and energy homeostasis. Consequently, Gloms prioritize immune-related adaptations, whereas PCT focus on oxidative stress recovery and metabolic reprogramming. These tissue-specific differences are further supported by our dimensional reduction analysis (see Fig 5, which shows PCA and t-SNE plots illustrating clear clustering of Gloms and PCT samples across experimental groups), where both PCA and t-SNE plots show clear clustering among WT, NKO, WT-STZ, and NKO-STZ groups. Notably, the distinct clustering observed within the glomerular samples indicates that different molecular mechanisms underlie the responses in Gloms compared to PCT, corroborating the pathway enrichment results.

### Suppression of adaptations under NQO1 deficiency (WT-STZ vs NKO-STZ)

**Fig 6:**
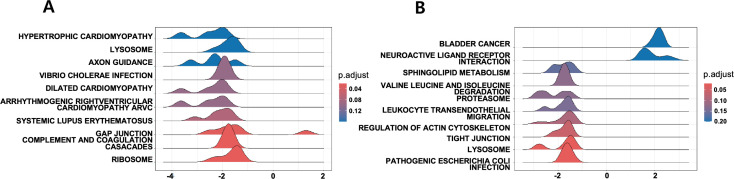
Ridge plots of the top 10 enriched KEGG pathways (q < 0.25) in WT-STZ vs. NKO-STZ comparisons. (A) Gloms and (B) PCT. The ridge plots display the distribution of normalized enrichment scores (NES) for enriched KEGG pathways with q-values < 0.25.

Comparisons between STZ-only and STZNKO conditions revealed a substantial decrease in pathways critical for adaptation to damage and immune response across both Gloms and PCT.(See Fig 6 for a visual representation of the top 10 representative pathways, with corresponding enrichment details summarized in Table 3 (Gloms) and Table 4 (PCT))

**Table 3:** Gene set enrichment analysis (GSEA) of glomeruli (Gloms) from WT-STZ vs. NKO-STZ (q < 0.25).

Pathway	Set Size	NES	P-value	P-adjust	Q-value	Core Gene Names
KEGG_RIBOSOME	9	−2.36	1.16E-03	5.94E-02	5.58E-02	Rpl9/Rps27/Rpl8/Rpl18/Rpl10a/Rps3a1/Rps3/Rps9
KEGG_COMPLEMENT_AND_COAGULATION_CASCADES	5	−2.28	4.85E-04	5.94E-02	5.58E-02	Cfh/Plau/C2/C1rb
KEGG_GAP_JUNCTION	7	−2.22	1.36E-03	5.94E-02	5.58E-02	Tubb6/Gnas/Gucy1a1/Pdgfrb/Prkaca/Pdgfd
KEGG_SYSTEMIC_LUPUS_ERYTHEMATOSUS	9	−2.11	4.83E-03	1.35E-01	1.27E-01	H3f3b/C2/C1rb/H4c8/H2az1/H4c17
KEGG_VIBRIO_CHOLERAE_INFECTION	4	−1.9	5.15E-03	1.35E-01	1.27E-01	Slc12a2/Actg1/Prkaca
KEGG_ARRHYTHMOGENIC_RIGHT_VENTRICULAR_CARDIOMYOPATHY_ARVC	5	−1.99	9.65E-03	1.77E-01	1.67E-01	Actg1/Cacnb1/Dmd/Cacna2d3
KEGG_DILATED_CARDIOMYOPATHY	9	−1.99	1.08E-02	1.77E-01	1.67E-01	Actg1/Prkaca/Cacnb1/Dmd/Cacna2d3
KEGG_NITROGEN_METABOLISM	2	1.62	1.08E-02	1.77E-01	1.67E-01	Car9/Cps1
KEGG_AXON_GUIDANCE	6	−1.84	1.35E-02	1.96E-01	1.84E-01	Dpysl2/Sema5a/Nrp1/Efnb1
KEGG_PATHOGENIC_ESCHERICHIA_COLI_INFECTION	4	−1.7	1.77E-02	2.29E-01	2.15E-01	Tubb6/Krt18/Actg1
KEGG_MELANOMA	3	−1.69	1.92E-02	2.29E-01	2.15E-01	Pdgfrb/Pdgfd
KEGG_HYPERTROPHIC_CARDIOMYOPATHY_HCM	7	−1.78	2.34E-02	2.47E-01	2.32E-01	Actg1/Cacnb1/Dmd/Cacna2d3
KEGG_LYSOSOME	11	−1.77	2.45E-02	2.47E-01	2.32E-01	Ctsh/Ctsd/Gba/Ctsa/Laptm4a/Psap/Man2b1

Significantly enriched KEGG pathways comparing WT-STZ and NKO-STZ glomeruli, with normalized enrichment score (NES), p-value, p-adjust, q-value, and representative genes.

**Abbreviations:** NES, normalized enrichment score; p-adjust, adjusted p-value; KEGG, Kyoto Encyclopedia of Genes and Genomes.

**Table 4:** Gene set enrichment analysis (GSEA) of proximal convoluted tubules (PCT) from WT-STZ vs. NKO-STZ (q < 0.25).

Pathway	Set Size	NES	P-value	P-adjust	Q-value	Core Gene Names
KEGG_PATHOGENIC_ESCHERICHIA_COLI_INFECTION	9	−2.43	2.60E-04	1.85E-02	1.64E-02	Arpc3/Ctnnb1/Actb/Rhoa/Wasl/Ywhaz/Arpc5/Ncl
KEGG_LYSOSOME	13	−2.32	3.59E-04	1.85E-02	1.64E-02	Hexb/Psap/Man2b1/Fuca1/Lamp2/Atp6v0c/Igf2r/Galc/Atp6v1h/Ctso/Ap3s1
KEGG_TIGHT_JUNCTION	7	−2.12	1.67E-03	5.74E-02	5.10E-02	Ctnnb1/Ppp2ca/Actb/Rhoa/Cldn10/Yes1
KEGG_REGULATION_OF_ACTIN_CYTOSKELETON	17	−2.09	3.54E-03	9.11E-02	8.10E-02	Arpc2/Arpc3/Mapk1/Braf/Actb/Rhoa/Abi2/Wasl/Arpc5/Fgf5/Ppp1cb/Itga4/Nckap1
KEGG_VALINE_LEUCINE_AND_ISOLEUCINE_DEGRADATION	4	−1.86	6.17E-03	1.27E-01	1.13E-01	Acad8/Dbt/Acads
KEGG_PROTEASOME	5	−1.92	7.48E-03	1.28E-01	1.14E-01	Psmd6/Psmc3/Psma2/Psmd14
KEGG_LEUKOCYTE_TRANSENDOTHELIAL_MIGRATION	6	−1.93	9.71E-03	1.43E-01	1.27E-01	Ctnnb1/Actb/Rhoa/Cldn10/Itga4
KEGG_SPHINGOLIPID_METABOLISM	7	−1.84	1.27E-02	1.64E-01	1.46E-01	Degs2/Degs1/Sgpl1/Galc/Plpp1/Sptlc2
KEGG_BLADDER_CANCER	7	1.77	1.94E-02	2.00E-01	1.78E-01	Fgfr3/Mapk3/E2f2/Rb1/Rps6ka5
KEGG_NEUROACTIVE_LIGAND_RECEPTOR_INTERACTION	19	1.73	1.83E-02	2.00E-01	1.78E-01	Gabrr2/Gabrg1/F2rl2/Chrna9/Avpr1b/Avpr2/Rxfp2/Gabrg2/Gabbr1/Lpar3/P2ry10/Nmbr/Oprm1/Chrnb1/F2rl3/Gm10334/Glp2r
KEGG_VIBRIO_CHOLERAE_INFECTION	6	−1.74	3.04E-02	2.68E-01	2.38E-01	Arf1/Actb/Atp6v0c/Pdia4/Atp6v1h
KEGG_PEROXISOME	6	−1.71	3.51E-02	2.68E-01	2.38E-01	Dhrs4/Pex11a/Pxmp2/Ephx2/Acox1
KEGG_PYRIMIDINE_METABOLISM	8	−1.68	3.89E-02	2.68E-01	2.38E-01	Dpys/Cda/Tk2/Polr1d/Nt5e/Nt5c3
KEGG_N_GLYCAN_BIOSYNTHESIS	7	−1.65	3.90E-02	2.68E-01	2.38E-01	Alg14/Rpn2/Alg9/Ddost/B4galt1/Mgat1
KEGG_FOCAL_ADHESION	14	−1.64	3.45E-02	2.68E-01	2.38E-01	Mapk1/Braf/Ctnnb1/Col1a2/Actb/Rhoa/Hgf/Ppp1cb/Itga4
KEGG_RIBOSOME	10	−1.68	4.16E-02	2.68E-01	2.38E-01	Rpl3/Rps29/Rps3a1/Rpl5/Rps9/Rpl9/Rps12/Rpl10

Significantly enriched KEGG pathways comparing WT-STZ and NKO-STZ PCT, listed with normalized enrichment score (NES), p-value, p-adjust, q-value, and representative genes.

**Abbreviations:** NES, normalized enrichment score; p-adjust, adjusted p-value; KEGG, Kyoto Encyclopedia of Genes and Genomes.

In Gloms, Ribosome (NES = −2.36), Complement/coagulation (−2.28), Gap junction (−2.22), and Systemic lupus erythematosus (−2.11) were markedly suppressed, indicating diminished protein synthesis, immune regulation, and cell–cell communication under NQO1 deficiency.

Similarly, PCT showed suppression in key adaptive pathways, including Pathogenic E. coli infection (−2.43), Lysosome (−2.32), Tight junction (−2.12), Actin cytoskeleton (−2.09), and Ribosome (−1.68), reflecting impaired inflammatory and structural responses. While modest activation of Neuroactive ligand–receptor interaction and Bladder cancer pathways was observed, these likely represent secondary or compensatory signals.

Overall, these findings demonstrate that NQO1 deficiency broadly impairs the kidney’s ability to manage oxidative stress and maintain essential adaptive processes, particularly those governing ribosomal activity, immune balance, and cytoskeletal integrity. The more pronounced suppression observed in Gloms—reflected by stronger downregulation of ribosomal biogenesis, complement/coagulation cascades—suggests a greater vulnerability of this compartment. In contrast, PCT retain a relatively higher capacity to sustain protein synthesis and metabolic adaptation despite NQO1 loss, although key structural and immune pathways remain impaired.

### PCT’s high adaptive capacity in NKO-STZ environment

When comparing WT mice to NKO-STZ mice, no pathways reached statistical significance in Gloms under the defined threshold (q < 0.25). Although the Peroxisome pathway (NES = −2.11, q = 0.29) had the lowest q-value, it did not meet the significance cutoff. This suggests a potential trend toward diminished peroxisomal function under NQO1 deficiency, but the lack of other significant pathways indicates a weaker transcriptional response in Gloms lacking NQO1.

In contrast, the PCT maintained robust activity in several key pathways even under NKO-STZ. Most notably, Ribosome (NES = +3.26) remained strongly active, indicating a sustained adaptive response that supports protein synthesis under heightened oxidative conditions. Upregulation of p53 signaling (NES = +2.08) implied ongoing cellular stress and a potential tilt toward apoptosis, while cancer-related pathways (NES = 1.8–1.9) underscored the delicate equilibrium between recovery and cell death.

Collectively, these findings highlight that although NQO1 deficiency impairs certain adaptive mechanisms in PCT, this compartment still exhibits a comparatively higher resilience and capacity to sustain metabolic and structural recovery than Gloms under STZ-induced diabetic stress. (see Supplementary Table S1 for full GSEA results).

### NKO vs. WT-STZ comparison

**Fig 7:**
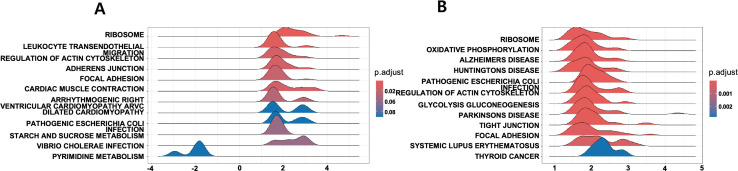
Ridge plots of top 10 enriched KEGG pathways for NQO1 vs. WT-STZ comparison. (A) Gloms and (B) PCT ridge plots display the top enriched KEGG pathways.

Although the WT-STZ and NKO groups are not perfectly matched controls, comparing them reveals how NQO1 deficiency alters responses to STZ-induced damage. Given that our analysis indicates NKO suppresses pathways otherwise activated by STZ, the contrast between these conditions may become more pronounced, thereby facilitating the detection of significant pathway changes in response to STZ. Previous reports indicate that, under certain conditions, NKO mice do not exhibit a marked increase in overall ROS after STZ treatment, suggesting that STZ-induced injury is not driven solely by global ROS elevation and may instead proceed through localized redox imbalances or alternative pathways [19]. Accordingly, our Gene Set Enrichment Analysis (GSEA) revealed markedly distinct pathway activations in Gloms and PCT between WT-STZ and NKO groups, with multiple pathways achieving exceptionally low *p* and *q* values. (see Fig 7, which shows ridge plots of the top 10 enriched KEGG pathways in NKO vs. WT-STZ comparisons for Gloms and PCT and see Supplementary Tables S2 and S3 for full results).

In Gloms, STZ treatment robustly upregulated the Ribosome (NES = +3.33, *P*-adjust = 1.02 × 10 ⁻ ⁶, q = 8.85 × 10 ⁻ ⁷), Leukocyte transendothelial migration (+2.52), and Regulation of Actin Cytoskeleton (+2.42) pathways—indicating active repair, immune mobilization, and cytoskeletal reorganization. Conversely, NKO Gloms featured higher Pyrimidine metabolism (NES = −1.87), suggesting an aberrant metabolic state possibly linked to impaired ROS clearance and intensified stress responses. (see Supplementary Table S2).

In PCT, STZ prominently activated multiple metabolic and structural pathways. The Ribosome pathway (NES = 5.07, *P*-adjust = 1.38 × 10 ⁻ ⁸, q = 1.14 × 10 ⁻ ⁸) showed the strongest upregulation, accompanied by Oxidative phosphorylation (NES = 3.71) and Glycolysis/Gluconeogenesis (NES = 2.99), reflecting a metabolic pivot toward energy production. Neurodegenerative-associated pathways (e.g., Alzheimer’s, Huntington’s, Parkinson’s) were also enriched, suggesting oxidative stress responses reminiscent of those found in neuronal cells. Cytoskeletal stability and cell adhesion were highlighted by Regulation of Actin Cytoskeleton (NES = 3.09), Tight Junction (2.94), and Focal Adhesion (2.75), while immune routes (Leukocyte transendothelial migration, SLE) and metabolic processes (TCA cycle, Glutathione metabolism, Pentose phosphate) completed a broad adaptive signature in STZ-treated PCT. (see Supplementary Table S3).

### Cytoskeletal changes under STZ damage: Gloms vs. PCT

A comparative analysis of cytoskeletal pathways in **NKO vs. WT-STZ** samples showed that PCT prominently engaged Actin Cytoskeleton (NES = 3.06), Focal Adhesion (NES = 2.75), and Tight Junction (NES = 3.03), reflecting the segment’s intense metabolic and transport requirements. (see Table 5, which compares cytoskeletal pathway enrichment between NKO and WT-STZ mice.)

**Table 5:** Gene set enrichment analysis (GSEA) of cytoskeletal pathways comparing NQO1 and WT-STZ.

Pathway	Gloms NES	Q-value	Core Gene Names	PCT NES	Q-value	Core Gene Names	NES Comparison
KEGG_REGULATION_OF_ACTIN_CYTOSKELETON	2.40	8.50E-03	Actg1/Arpc3/Wasf2/Slc9a1/Fn1/Ptk2/Iqgap1/Mapk1/Rhoa/Actb/Ppp1ca/Itgav/Itgb5/Fgf7/Rac1/Araf	3.06	1.22E-05	Tmsb4x/Cfl1/Ppp1cb/Cdc42/Arpc5/Map2k1/Msn/Rhoa/Actn4/Itgb6/Ezr/F2r/Rock2/Diaph1/Actb/Actg1/Cfl2/Nckap1/Itgb5/Pfn1/Rras2/Myl12a/Pdgfb/Itga1	PCT > Gloms
KEGG_TIGHT_JUNCTION	1.81	8.72E-02	Actg1/Tjp1/Rhoa/Ctnnb1/Exoc4/Actb/Magi2/Ctnna1	3.03	1.22E-05	Exoc4/Cdc42/Ctnnb1/Ppp2ca/Gnai2/Rhoa/Actn4/Epb41/Pard3/Actb/Actg1/Ppp2r1b/Ppp2r1a/Rras2/Myl12a	PCT > Gloms
KEGG_FOCAL_ADHESION	2.23	1.71E-02	Actg1/Fn1/Spp1/Ptk2/Mapk1/Rhoa/Col4a1/Col4a2/Ctnnb1/Actb/Ppp1ca/Itgav/Itgb5/Flna/Rac1	2.75	2.64E-04	Col4a1/Parva/Ppp1cb/Cdc42/Spp1/Flt1/Map2k1/Ctnnb1/Rhoa/Actn4/Itgb6/Mapk8/Rock2/Diaph1/Actb/Actg1/Itgb5/Col1a2/Myl12a/Pdgfb/Itga1/Capn2/Jun/Pak1/Col4a2	PCT > Gloms
KEGG_ADHERENS_JUNCTION	2.37	1.34E-02	Actg1/Wasf2/Tjp1/Iqgap1/Mapk1/Ptprb/Rhoa/Ctnnb1/Actb/Ptprj/Ctnna1	1.99	3.52E-02	Cdc42/Ctnnb1/Rhoa/Actn4/Cdh1/Pard3/Actb/Actg1	Gloms > PCT

KEGG cytoskeletal pathways enriched in glomeruli (Gloms) and proximal convoluted tubules (PCT) when comparing NKO to WT-STZ, listed with normalized enrichment score (NES), q-value, and representative genes. Actin cytoskeleton, tight junction, and focal adhesion pathways were more enriched in PCT, while adherens junction pathway was more enriched in glomeruli, indicating compartment-specific cytoskeletal differences between NKO and WT-STZ.

**Abbreviations:** NES, normalized enrichment score; q-value, false discovery rate; KEGG, Kyoto Encyclopedia of Genes and Genomes; Gloms, glomeruli; PCT, proximal convoluted tubules.

**Fig 8:**
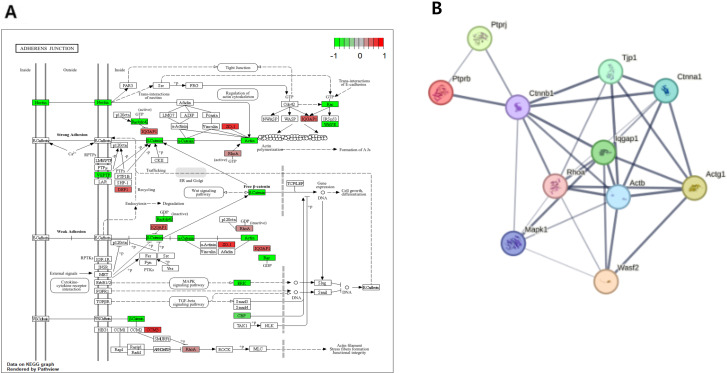
Enrichment of adherens junction pathway and protein interaction network in gloms (a) KEGG pathway visualization of the enriched Adherens Junction pathway in Gloms. (b) STRING network analysis of key Adherens Junction proteins identified in NKO vs. WT-STZ Gloms. The network illustrates the functional associations among proteins encoded by Ptprj, Actb, Tjp1, Ctnna1, Rhoa, Iqgap1, Wasf2, Ctnnb1, Actg1, and Ptprb, which are involved in cell–cell adhesion and cytoskeletal regulation. Each node represents all isoforms produced by a single protein-coding gene locus. Colored nodes indicate query proteins and their first-shell interactors, while white nodes represent second-shell interactors. Filled nodes denote proteins with known or predicted 3D structures. Edges signify functional protein-protein associations rather than direct physical interactions, with thicker and more saturated edges reflecting higher confidence scores. This network highlights the coordinated role of these proteins in maintaining glomerular podocyte integrity and actin cytoskeleton dynamics under STZ-induced diabetic stress.

By contrast, the Adherens Junction pathway (NES = 2.37) was more pronounced in Gloms, emphasizing the necessity of stabilizing podocyte foot processes and preserving filtration barrier integrity. Key upregulated genes included *Actg1*, *Ctnna1*, *Tjp1*, *Rhoa*, and *Iqgap1*, collectively reinforcing cell–cell adhesion, cytoskeletal remodeling, and resistance to mechanical stress. (See Fig 8. for a visual representation of glomerular cytoskeletal adaptations and the enrichment of the Adherens Junction pathway, along with key protein–protein interaction networks involved in podocyte structure) Further underscoring this emphasis on Adherens Junctions, an additional KEGG analysis in Gloms (NKO vs. STZNKO) identified Adherens Junction (NES = 1.90, *p* = 6.0 × 10 ⁻^3^, *q* = 2.60 × 10 ⁻ ¹) as the most prominently enriched pathway. The genes contributing to this enrichment (e.g., *Actn4*, *Ctnnb1*, *Rhoa*, *Wasf2*, *Ctnnd1*, *Ctnna1*, *Ptprb*) encode proteins pivotal for maintaining cell–cell adhesion and supporting podocyte foot processes. This finding underscores the structural and functional relevance of adherens junctions in Gloms, particularly under STZ-induced diabetic stress, and contrasts with the different cytoskeletal dynamics observed in PCT.

Collectively, these findings illustrate distinct cytoskeletal strategies in response to STZ damage: PCT prioritize dynamic cytoskeletal rearrangements and metabolic resilience to sustain reabsorption, whereas Gloms fortify robust adhesion complexes to protect the filtration barrier from both mechanical forces and ROS-induced challenges.

## Discussion

Our findings indicate that STZ-induced diabetic injury triggers notable ribosome activation, p53 signaling, immune/inflammatory responses, and cytoskeletal remodeling across Gloms and PCT. The responses differed between compartments: Gloms exhibit heightened immune and p53-associated pathways, whereas PCT show robust oxidative phosphorylation, glutathione metabolism, and glycolysis/gluconeogenesis, suggesting partially distinct adaptive routes under hyperglycemia. Both compartments exhibited significant upregulation of the ribosome pathway, which can be interpreted as an adaptive response to hyperglycemic stress, allowing cells to sustain protein synthesis despite metabolic challenges.[25]

A central observation is that NQO1 deficiency in the STZ model compromises key adaptive pathways such as protein synthesis, immune/inflammatory regulation, and cytoskeletal reorganization, and imposes metabolic imbalances. While some ischemia/reperfusion models have shown that lack of NQO1 can heighten ROS and exacerbate renal damage, [26] other data in STZ models suggest that global ROS may not uniformly surge in NKO mice, [24] implying localized or alternative protective roles for NQO1. Indeed, our data support a broader function of NQO1 that extends beyond controlling total ROS levels, including the maintenance of redox equilibrium within specific compartments. NQO1 is a cytoprotective flavoprotein that reduces quinones to hydroquinones using NADH, thereby restoring the NAD ⁺ /NADH balance and buffering the redox state under diabetic or oxidative stress. Given that PCT cells depend on mitochondrial metabolism and podocytes are highly sensitive to redox imbalance, NQO1 loss may exacerbate stress by shifting the NAD ⁺ /NADH ratio toward a more reduced state. NQO1 is well-known for its antioxidant function. It detoxifies reactive quinones and limits oxidative stress by preventing redox cycling, thereby reducing intracellular ROS levels. In this regard, part of NQO1’s protective role in hyperglycemia is clearly ROS-dependent, particularly in preventing ROS-induced damage to glomerular and tubular cells. However, emerging evidence also supports ROS-independent functions of NQO1, especially through its role in maintaining NAD + /NADH homeostasis, regulating cellular metabolism, and stabilizing transcription factors (e.g., NRF2, p53) via protein-protein interactions. Therefore, the role of NQO1 in STZ-induced DKD animal model may involve both ROS-dependent and ROS-independent mechanisms, which synergistically contribute to cellular protection in diabetic kidneys.These findings are in line with reports of NQO1 upregulation in hyperglycemic Gloms, notably in podocytes, where it can defend against oxidative insults and preserve cytoskeletal organization.[10] In our STZ model, NQO1 deficiency suppressed the previously upregulated ribosome and immune/inflammatory pathways in Gloms, implying that local redox stabilization by NQO1 helps maintain essential functions like protein synthesis.

Interestingly, a recent neuronal study demonstrated that NQO1 modulates redox balance in a context-dependent manner: knockdown NQO1 enhanced glutathione defenses and reduced ferroptosis, whereas overexpression improved mitochondrial toxin tolerance by elevating NAD ⁺.[27] These findings suggest that NQO1 influences cellular metabolism by regulating oxidative stress pathways, which may also apply to renal compartments. Given this metabolic role, differences in oxidative stress responses between Gloms and PCT could contribute to their distinct adaptive capacities under NQO1 deficiency.

A similar principle may hold in PCT, where STZ led to enhanced oxidative phosphorylation, glutathione metabolism, and other metabolic reprogramming, supporting adaptive responses to hyperglycemia. However, NQO1 deficiency resulted in the suppression of immune, cytoskeletal, and metabolic pathways, while ribosome activity was comparatively less suppressed than in Gloms. This relative difference may be linked to PCT’s ability to maintain oxidative phosphorylation and antioxidant defenses, which could help sustain protein synthesis despite NQO1 loss. In contrast, Gloms exhibited more pronounced ribosomal suppression under NQO1 deficiency, potentially reflecting greater metabolic vulnerability. Future analyses of compartment-specific oxidative stress markers (e.g., mitochondrial function, GSH/GSSG ratios) will be necessary to determine whether these metabolic differences underlie the distinct ribosome regulation patterns observed in Gloms and PCT.

Our GSEA also highlighted the importance of cytoskeletal frameworks in each region: PCT predominantly engage Actin Cytoskeleton, Tight Junction, and Focal Adhesion modules to meet high transport demands, while glomerular cells rely more on the Adherens Junction pathway for stabilizing podocyte foot processes and maintaining filtration integrity. Within the adherens junction pathway, key genes such as Actg1, Ctnna1, Tjp1, Rhoa, and Iqgap1 appear integral to podocyte structure, underscoring how cytoskeletal integrity is linked to glomerular function under diabetic stress. Notably, NQO1 deficiency may undermine these cytoskeletal defenses, although quantifying how much more the glomerulus is affected compared to PCT may require direct functional assays.

A key limitation of this study is the lack of protein-level validation for the transcriptomic findings. While we identified significant pathway alterations, we did not confirm whether these changes translate into corresponding protein expression or functional effects. Our study employed an STZ-induced diabetic model, which primarily reflects acute or early-stage renal responses to hyperglycemia rather than the chronic, fibrotic progression seen in advanced human DKD. Thus, while the model is useful for exploring early redox dysregulation and compartment-specific stress pathways, caution is warranted in extrapolating these findings to later disease stages. Furthermore, STZ can exert systemic toxicity beyond hyperglycemia, including ROS generation, which may confound renal injury mechanisms. To mitigate this, we applied a moderate dosing regimen with a stabilization period to ensure hyperglycemia-driven effects. Nonetheless, these limitations should be considered when interpreting the mechanistic relevance of our transcriptomic findings. Additionally, we expanded our pathway enrichment analysis beyond KEGG to include Reactome and GO:BP, which resulted in higher set sizes and lower q-values. However, the inclusion of numerous small pathways made interpretation more complex, as many overlapped with KEGG pathways. To ensure consistency and reliability, we focused on KEGG pathways that aligned with findings from other databases while omitting extensive additional pathways. This highlights a limitation in our study, as we did not provide a comprehensive report of all identified pathways. Nonetheless, the strong overlap between KEGG-enriched pathways and those from alternative databases supports the robustness of our KEGG-based interpretation. Future studies integrating proteomic validation and broader pathway-level analyses will be essential for confirming these transcriptomic changes and their functional implications.

## Conclusion

Our study demonstrates the compartment-specific impact of NQO1 deficiency under STZ-induced hyperglycemia, providing new insight into its functional roles in diabetic kidneys. In Gloms from NKO mice, the typical STZ-induced activation of ribosome biogenesis and immune pathways was significantly suppressed, indicating impaired protein synthesis and immune regulation. In PCT, NQO1 deficiency led to the downregulation of immune, cytoskeletal, and metabolic pathways with ribosomal activity remaining relatively preserved. Notably, the adherens junction pathway was prominently activated in Gloms, suggesting a compensatory mechanism to preserve podocyte foot process integrity under stress. These findings collectively highlight NQO1 as a critical regulator of compartment-specific adaptive responses, essential for maintaining proteostasis, immune balance, and cytoskeletal architecture in the diabetic kidney. Therefore, Our findings highlight NQO1 as a compartment-specific therapeutic target, suggesting that strategies such as activating glomerular NQO1 to protect podocyte integrity and modulating tubular NQO1 to enhance metabolic adaptation could be effective interventions.

## Supporting information

S1 Table Gene set enrichment analysis (GSEA) of proximal convoluted tubules (PCT) comparing WT and STZNKO. Pathways with q < 0.25 are listed with NES and associated genes.
(DOCX)

S2 Table GSEA of glomeruli comparing NKO and STZ-treated mice. Pathways with q < 0.25 are listed with NES and associated genes.
(DOCX)

S3 Table GSEA of PCT comparing NKO and STZ-treated mice. Pathways with q < 0.25 are listed with NES and associated genes.
(DOCX)

S1 Fig Region of interest (ROI) analysis for spatial transcriptomic profiling. (A, D) Representative kidney sections showing ROIs selected based on tissue morphology, including both injured and intact regions. Staining for CD10, CD31, PanCK, and nuclear dyes guided ROI selection, refined by gene markers (Krt8, Mme, DNA). (B, C, E, F) Quantification of Krt8 (B, E) and Mme (C, F) expression in ROIs, normalized using Q3, illustrating spatial gene expression variation.
(TIFF)

S1 File Raw data of differentially expressed gene analysis (Glomerulus & Proximal Tubules) and mouse parameters (albumin creatinine ratio, fast blood glucose, foot process width, glomerular basement membrane thickness). (ZIP)

S2 File Animal IRB approval document. (PDF)
