# Supplementary material for: Compartment-specific adaptive responses and dysregulation under NQO1 deficiency in diabetic kidney disease: A transcriptomic GSEA-based investigation
Source: PLoS One. 2025 Sep 8;20(9):e0331582. doi: 10.1371/journal.pone.0331582 (PMC12416748; doi:10.1371/journal.pone.0331582)
Supplement: S1 Table — Pathways with q < 0.25 are listed with NES and associated genes. (DOCX) [file pone.0331582.s001.docx]

**S1 Table. PCT WT vs. STZNKO GSEA Analysis (q-value < 0.25)**

| **Pathway** | **Set Size** | **NES** | **P-value** | **P-adjust** | **Q-value** | **Core Gene Names** |
| --- | --- | --- | --- | --- | --- | --- |
| KEGG_RIBOSOME | 28 | 3.26 | 4.68E-09 | 7.48E-07 | 7.28E-07 | Rpl7a/Rpl28/Rpl37/Rpl27/Rpl27a/Rps7/Rpl38/Rpl13a/Rps3/Rpl24/Rpl36/Rpl10a/Rps4x/Rps2/Rpl14/Rps23/Rps10/Rpl31/Rpl19/Rps27l/Rpl13/Rpl22/Rpl4/Rps13 |
| KEGG_P53_SIGNALING_PATHWAY | 10 | 2.08 | 1.95E-03 | 1.56E-01 | 1.52E-01 | Ccng1/Mdm2/Zmat3/Ccnd1/Sesn3/Cdkn1a/Rchy1 |
| KEGG_CHRONIC_MYELOID_LEUKEMIA | 8 | 1.88 | 7.87E-03 | 2.26E-01 | 2.20E-01 | Mdm2/Mapk3/Cdkn1b/Ccnd1/Cdkn1a |
| KEGG_PROSTATE_CANCER | 9 | 1.85 | 7.29E-03 | 2.26E-01 | 2.20E-01 | Mdm2/Mapk3/Cdkn1b/Ccnd1/Cdkn1a/Creb1 |
| KEGG_GAP_JUNCTION | 7 | 1.82 | 8.18E-03 | 2.26E-01 | 2.20E-01 | Adcy6/Mapk3/Tuba1b/Gnai2/Gucy1a2/Kras/Grm5 |
| KEGG_BLADDER_CANCER | 6 | 1.8 | 8.49E-03 | 2.26E-01 | 2.20E-01 | Mdm2/Mapk3/Ccnd1/Cdkn1a |
