# Supplementary material for: Compartment-specific adaptive responses and dysregulation under NQO1 deficiency in diabetic kidney disease: A transcriptomic GSEA-based investigation
Source: PLoS One. 2025 Sep 8;20(9):e0331582. doi: 10.1371/journal.pone.0331582 (PMC12416748; doi:10.1371/journal.pone.0331582)
Supplement: S2 Table — Pathways with q < 0.25 are listed with NES and associated genes. (DOCX) [file pone.0331582.s002.docx]

**S2 Table. Glomeruli (Gloms) NKO vs. STZ GSEA Analysis (q-value < 0.25)**

| **Pathway** | **Set Size** | **NES** | **P-value** | **P-adjust** | **Q-value** | **Core Gene Names** |
| --- | --- | --- | --- | --- | --- | --- |
| KEGG_RIBOSOME | 21 | 3.33 | 7.31E-09 | 1.02E-06 | 8.85E-07 | Rps24/Rps3/Rpl34/Rps9/Rps3a1/Rps27a/Rps27/Rps7/Rplp1/Rpl31/Rps23/Rps26/Rpl23/Rpl37/Rpl22 |
| KEGG_LEUKOCYTE_TRANSENDOTHELIAL_MIGRATION | 10 | 2.52 | 5.33E-05 | 3.71E-03 | 3.23E-03 | Actg1/Ptk2/Rhoa/Ctnnb1/Cxcr4/Actb/Ptpn11/Ctnna1/Cxcl12/Rac1 |
| KEGG_REGULATION_OF_ACTIN_CYTOSKELETON | 20 | 2.42 | 2.51E-04 | 1.04E-02 | 9.04E-03 | Actg1/Arpc3/Wasf2/Slc9a1/Fn1/Ptk2/Iqgap1/Mapk1/Rhoa/Actb/Ppp1ca/Itgav/Itgb5/Fgf7/Rac1/Araf |
| KEGG_ADHERENS_JUNCTION | 15 | 2.31 | 3.25E-04 | 1.04E-02 | 9.04E-03 | Actg1/Wasf2/Tjp1/Iqgap1/Mapk1/Ptprb/Rhoa/Ctnnb1/Actb/Ptprj/Ctnna1 |
| KEGG_CARDIAC_MUSCLE_CONTRACTION | 6 | 2.21 | 3.73E-04 | 1.04E-02 | 9.04E-03 | Atp1b3/Cacna2d3/Slc9a1/Atp1a1/Cacnb1/Cox6b1 |
| KEGG_FOCAL_ADHESION | 20 | 2.25 | 7.05E-04 | 1.63E-02 | 1.42E-02 | Actg1/Fn1/Spp1/Ptk2/Mapk1/Rhoa/Col4a1/Col4a2/Ctnnb1/Actb/Ppp1ca/Itgav/Itgb5/Flna/Rac1 |
| KEGG_ARRHYTHMOGENIC_RIGHT_VENTRICULAR_CARDIOMYOPATHY_ARVC | 9 | 2.11 | 1.22E-03 | 2.42E-02 | 2.11E-02 | Actg1/Cacna2d3/Ctnnb1/Actb/Cacnb1/Ctnna1/Itgav/Itgb5 |
| KEGG_STARCH_AND_SUCROSE_METABOLISM | 4 | 1.94 | 2.41E-03 | 3.72E-02 | 3.24E-02 | Ugp2/Pygb/Gpi1/Gaa |
| KEGG_VIBRIO_CHOLERAE_INFECTION | 4 | 1.93 | 2.41E-03 | 3.72E-02 | 3.24E-02 | Actg1/Gnas/Tjp1/Actb |
| KEGG_DILATED_CARDIOMYOPATHY | 8 | 1.99 | 5.63E-03 | 7.15E-02 | 6.23E-02 | Actg1/Gnas/Cacna2d3/Actb/Cacnb1/Itgav/Itgb5 |
| KEGG_PATHOGENIC_ESCHERICHIA_COLI_INFECTION | 6 | 1.94 | 6.17E-03 | 7.15E-02 | 6.23E-02 | Actg1/Arpc3/Rhoa/Ctnnb1/Actb |
| KEGG_PYRIMIDINE_METABOLISM | 5 | -1.87 | 6.04E-03 | 7.15E-02 | 6.23E-02 | Entpd4/Dut/Nme6/Entpd6 |
| KEGG_NEUROTROPHIN_SIGNALING_PATHWAY | 6 | 1.92 | 6.95E-03 | 7.43E-02 | 6.47E-02 | Mapkapk2/Mapk1/Rhoa/Ptpn11/Rac1/Calm1 |
| KEGG_COLORECTAL_CANCER | 5 | 1.82 | 8.18E-03 | 8.12E-02 | 7.07E-02 | Mapk1/Rhoa/Ctnnb1/Rac1/Araf |
| KEGG_AMINO_SUGAR_AND_NUCLEOTIDE_SUGAR_METABOLISM | 3 | 1.7 | 9.88E-03 | 9.15E-02 | 7.97E-02 | Gne/Ugp2/Gpi1 |
| KEGG_MAPK_SIGNALING_PATHWAY | 15 | 1.79 | 1.17E-02 | 1.01E-01 | 8.83E-02 | Mapkapk2/Cacna2d3/Stk3/Mapk1/Fas/Cacnb1/Map4k4/Flna/Fgf7/Rac1/Ppp5c |
| KEGG_FC_GAMMA_R_MEDIATED_PHAGOCYTOSIS | 6 | 1.81 | 1.29E-02 | 1.05E-01 | 9.17E-02 | Arpc3/Wasf2/Asap1/Mapk1 |
| KEGG_TIGHT_JUNCTION | 11 | 1.78 | 1.36E-02 | 1.05E-01 | 9.17E-02 | Actg1/Tjp1/Rhoa/Ctnnb1/Exoc4/Actb/Magi2/Ctnna1 |
| KEGG_SPLICEOSOME | 11 | 1.77 | 1.48E-02 | 1.08E-01 | 9.43E-02 | Dhx8/Ppil1/Hnrnpu/Pqbp1/Snrpd3/Sf3b4/Slu7 |
| KEGG_HYPERTROPHIC_CARDIOMYOPATHY_HCM | 9 | 1.74 | 1.59E-02 | 1.10E-01 | 9.60E-02 | Actg1/Cacna2d3/Actb/Cacnb1/Itgav/Prkab2/Itgb5 |
| KEGG_AXON_GUIDANCE | 13 | 1.78 | 1.68E-02 | 1.11E-01 | 9.70E-02 | Sema6d/Ptk2/Mapk1/Rhoa/Cxcr4/Ntn4/Dpysl2/Nrp1/Cxcl12/Rac1 |
| KEGG_ENDOMETRIAL_CANCER | 4 | 1.66 | 3.12E-02 | 1.97E-01 | 1.72E-01 | Mapk1/Ctnnb1/Ctnna1/Araf |
| KEGG_ENDOCYTOSIS | 13 | 1.65 | 3.86E-02 | 2.33E-01 | 2.03E-01 | Asap1/Ehd2/Chmp1b/Nedd4/Ap2a2/Cxcr4/Chmp4b/Csf1r/Sh3glb1/Arfgap2 |
| KEGG_HUNTINGTONS_DISEASE | 9 | 1.59 | 4.26E-02 | 2.47E-01 | 2.15E-01 | Sp1/Dlg4/Ap2a2/Ppid/Cox6b1/Dnah1 |
