# Supplementary material for: Compartment-specific adaptive responses and dysregulation under NQO1 deficiency in diabetic kidney disease: A transcriptomic GSEA-based investigation
Source: PLoS One. 2025 Sep 8;20(9):e0331582. doi: 10.1371/journal.pone.0331582 (PMC12416748; doi:10.1371/journal.pone.0331582)
Supplement: S3 Table — Pathways with q < 0.25 are listed with NES and associated genes. (DOCX) [file pone.0331582.s003.docx]

**S3 Table. Proximal Convoluted Tubules (PCT) NKO vs. STZ GSEA Analysis (q-value < 0.25)**

| **Pathway** | **Set Size** | **NES** | **P-value** | **P-adjust** | **Q-value** | **Core Gene Names** |
| --- | --- | --- | --- | --- | --- | --- |
| KEGG_RIBOSOME | 61 | 5.07 | 1.00E-10 | 1.38E-08 | 1.14E-08 | Rps19/Rpl12/Rpl14/Rpl15/Rpl17/Rpl38/Rpl31/Rpl13a/Rpl18/Rpl10a/Fau/Rps26/Rpl27a/Rpl35/Rps21/Rpl35a/Rps7/Rps20/Rpl6/Rps24/Rps2/Rps4x/Rpl10/Rpl37/Rps15/Rpl9/Rplp0/Rps13/Rps3a1/Rps8/Rps15a/Rps17/Rps23/Rpl5/Rpl18a/Rps27/Rpl26/Rps11/Rps9/Rps27a/Rps10/Rpl13/Rpl23/Rplp1/Rpl23a/Rps25/Rps29/Rpsa/Rps3/Rps27l/Rpl4/Rps16/Rps12/Rpl11/Rpl27/Rpl19/Rps28/Rpl37a/Rps6/Rpl8 |
| KEGG_OXIDATIVE_PHOSPHORYLATION | 35 | 3.71 | 1.57E-10 | 1.38E-08 | 1.14E-08 | Cox5b/Atp5o/Atp5pb/Atp5g3/Sdhd/Ndufb6/Atp5k/Atp5b/Ndufs5/Ndufa5/Uqcrh/Cox4i1/Atp5h/Ndufs3/Atp5a1/Atp6ap1/Uqcrb/Uqcrfs1/Atp6v1e1/Cox6b2/Ndufb1/Cox7b/Ndufa9/Atp6v0c/Atp5e/Atp5j2/Uqcrc2/Ndufv2/Atp6v0e/Atp6v1b2/Ndufs1/Atp4a |
| KEGG_ALZHEIMERS_DISEASE | 37 | 3.52 | 4.92E-10 | 2.87E-08 | 2.38E-08 | Cox5b/Atp5o/Atp5pb/Atp5g3/Sdhd/Ndufb6/Atp5b/Ndufs5/Ndufa5/Uqcrh/Cox4i1/App/Atp5h/Hsd17b10/Ndufs3/Atp5a1/Calm1/Apoe/Uqcrb/Uqcrfs1/Cox6b2/Ndufb1/Cox7b/Ndufa9/Gapdh/Atp5e/Capn2/Uqcrc2/Ndufv2/Calm2 |
| KEGG_HUNTINGTONS_DISEASE | 37 | 3.39 | 3.01E-09 | 1.32E-07 | 1.09E-07 | Cox5b/Atp5o/Atp5pb/Clta/Atp5g3/Sdhd/Ndufb6/Atp5b/Polr2f/Ndufs5/Ndufa5/Uqcrh/Cox4i1/Atp5h/Ndufs3/Ap2b1/Atp5a1/Uqcrb/Uqcrfs1/Cox6b2/Ndufb1/Cox7b/Ndufa9/Atp5e/Vdac3/Uqcrc2/Ndufv2/Nrf1/Gpx1/Slc25a4/Ndufs1/Tgm2 |
| KEGG_PATHOGENIC_ESCHERICHIA_COLI_INFECTION | 14 | 3.12 | 4.11E-08 | 1.44E-06 | 1.20E-06 | Cdc42/Tuba4a/Arpc5/Tubb4b/Ctnnb1/Rhoa/Cdh1/Tuba1b/Ezr/Rock2/Actb/Actg1 |
| KEGG_GLYCOLYSIS_GLUCONEOGENESIS | 19 | 2.99 | 2.97E-07 | 8.67E-06 | 7.20E-06 | Pgk1/Pgam1/Eno1/Pkm/Tpi1/Pklr/Akr1a1/Ldhb/Aldoa/Aldh2/Aldh7a1/Aldob/Aldh3b1/Gapdh/Galm/Gpi1 |
| KEGG_REGULATION_OF_ACTIN_CYTOSKELETON | 35 | 3.09 | 5.63E-07 | 1.33E-05 | 1.10E-05 | Tmsb4x/Cfl1/Ppp1cb/Cdc42/Arpc5/Map2k1/Msn/Rhoa/Actn4/Itgb6/Ezr/F2r/Rock2/Diaph1/Actb/Actg1/Cfl2/Nckap1/Itgb5/Pfn1/Rras2/Myl12a/Pdgfb/Itga1 |
| KEGG_TIGHT_JUNCTION | 19 | 2.94 | 6.08E-07 | 1.33E-05 | 1.10E-05 | Exoc4/Cdc42/Ctnnb1/Ppp2ca/Gnai2/Rhoa/Actn4/Epb41/Pard3/Actb/Actg1/Ppp2r1b/Ppp2r1a/Rras2/Myl12a |
| KEGG_PARKINSONS_DISEASE | 36 | 2.94 | 2.26E-06 | 4.39E-05 | 3.65E-05 | Park7/Cox5b/Atp5o/Atp5pb/Atp5g3/Sdhd/Ndufb6/Atp5b/Ndufs5/Ndufa5/Uqcrh/Cox4i1/Atp5h/Ndufs3/Atp5a1/Uqcrb/Uqcrfs1/Cox6b2/Ndufb1/Cox7b/Ndufa9/Atp5e/Vdac3/Uqcrc2/Ndufv2 |
| KEGG_FOCAL_ADHESION | 34 | 2.75 | 1.03E-05 | 1.80E-04 | 1.50E-04 | Col4a1/Parva/Ppp1cb/Cdc42/Spp1/Flt1/Map2k1/Ctnnb1/Rhoa/Actn4/Itgb6/Mapk8/Rock2/Diaph1/Actb/Actg1/Itgb5/Col1a2/Myl12a/Pdgfb/Itga1/Capn2/Jun/Pak1/Col4a2 |
| KEGG_SYSTEMIC_LUPUS_ERYTHEMATOSUS | 17 | 2.68 | 1.90E-05 | 3.02E-04 | 2.50E-04 | H2-DMb1/C1qa/H4c17/H3c2/H3c1/H2-Aa/H4c8/C2/Actn4/H3f3b/H2-Ab1/Macroh2a1/C3/C1qb |
| KEGG_THYROID_CANCER | 6 | 2.32 | 1.91E-04 | 2.79E-03 | 2.31E-03 | Myc/Tpm3/Map2k1/Ctnnb1/Cdh1 |
| KEGG_CARDIAC_MUSCLE_CONTRACTION | 13 | 2.35 | 3.52E-04 | 4.49E-03 | 3.73E-03 | Cox5b/Tpm3/Atp1b3/Uqcrh/Cox4i1/Uqcrb/Uqcrfs1/Cox6b2/Tpm4/Cox7b/Uqcrc2 |
| KEGG_PENTOSE_PHOSPHATE_PATHWAY | 7 | 2.34 | 3.59E-04 | 4.49E-03 | 3.73E-03 | Tkt/Pgd/Aldoa/Aldob/Gpi1/Prps2/Rpia |
| KEGG_LEUKOCYTE_TRANSENDOTHELIAL_MIGRATION | 16 | 2.23 | 7.75E-04 | 9.04E-03 | 7.51E-03 | Cdc42/Ctnnb1/Gnai2/Msn/Rhoa/Actn4/Ezr/Rock2/Actb/Actg1 |
| KEGG_NEUROTROPHIN_SIGNALING_PATHWAY | 16 | 2.18 | 1.01E-03 | 1.10E-02 | 9.16E-03 | Ywhae/Map3k1/Cdc42/Map2k1/Arhgdia/Ywhah/Rhoa/Mapk8/Calm1 |
| KEGG_MISMATCH_REPAIR | 2 | -1.63 | 3.88E-03 | 4.00E-02 | 3.32E-02 | Rpa3 |
| KEGG_CITRATE_CYCLE_TCA_CYCLE | 8 | 1.99 | 4.69E-03 | 4.56E-02 | 3.79E-02 | Cs/Sdhd/Idh3g/Acly/Mdh2/Idh1/Idh3a |
| KEGG_LONG_TERM_DEPRESSION | 11 | 1.89 | 5.43E-03 | 5.00E-02 | 4.15E-02 | Gucy1a1/Map2k1/Ppp2ca/Gnai2/Ppp2r1b/Ppp2r1a/Pla2g12a |
| KEGG_ANTIGEN_PROCESSING_AND_PRESENTATION | 16 | 1.91 | 6.66E-03 | 5.82E-02 | 4.83E-02 | Psme2/H2-DMb1/Ctss/H2-Aa/Cd74/H2-D1/Hspa5/Ifi30/H2-Ab1 |
| KEGG_ADHERENS_JUNCTION | 13 | 1.93 | 9.04E-03 | 7.17E-02 | 5.95E-02 | Cdc42/Ctnnb1/Rhoa/Actn4/Cdh1/Pard3/Actb/Actg1 |
| KEGG_AXON_GUIDANCE | 17 | 1.89 | 9.42E-03 | 7.17E-02 | 5.95E-02 | Cfl1/Cdc42/Gnai2/Rhoa/Rock2/Srgap1/Dpysl2/Rhod/Cfl2/Sema5a |
| KEGG_LIMONENE_AND_PINENE_DEGRADATION | 3 | 1.76 | 8.87E-03 | 7.17E-02 | 5.95E-02 | Hadha/Aldh2/Aldh7a1 |
| KEGG_ECM_RECEPTOR_INTERACTION | 11 | 1.78 | 9.90E-03 | 7.22E-02 | 5.99E-02 | Col4a1/Spp1/Hspg2/Itgb6/Itgb5/Col1a2/Itga1 |
| KEGG_CELL_CYCLE | 12 | 1.94 | 1.09E-02 | 7.61E-02 | 6.32E-02 | Cdkn1a/Ywhae/Myc/Ywhah/Mdm2 |
| KEGG_OOCYTE_MEIOSIS | 15 | 1.89 | 1.27E-02 | 8.22E-02 | 6.82E-02 | Ywhae/Ppp1cb/Map2k1/Ywhah/Ppp2ca/Calm1/Ppp2r1b/Ppp2r1a/Anapc11 |
| KEGG_BLADDER_CANCER | 8 | 1.86 | 1.25E-02 | 8.22E-02 | 6.82E-02 | Cdkn1a/Myc/Map2k1/Mdm2/Cdh1 |
| KEGG_CHRONIC_MYELOID_LEUKEMIA | 8 | 1.84 | 1.37E-02 | 8.55E-02 | 7.10E-02 | Cdkn1a/Myc/Map2k1/Mdm2 |
| KEGG_GLUTATHIONE_METABOLISM | 12 | 1.89 | 1.44E-02 | 8.70E-02 | 7.22E-02 | Gsta5/Mgst1/Gsta3/Pgd/Mgst3/Gsto1/Idh1/Gpx1/Gstm5/Gstp1 |
| KEGG_LYSOSOME | 17 | 1.8 | 1.66E-02 | 9.63E-02 | 8.00E-02 | Igf2r/Ctss/Clta/Ctsd/Laptm4a/Cd63/Lamp2/Atp6ap1 |
| KEGG_AMINO_SUGAR_AND_NUCLEOTIDE_SUGAR_METABOLISM | 4 | 1.73 | 1.71E-02 | 9.63E-02 | 8.00E-02 | Npl/Gpi1/Hexa/Gfpt1 |
| KEGG_PROPANOATE_METABOLISM | 5 | 1.75 | 1.90E-02 | 1.04E-01 | 8.64E-02 | Hadha/Ldhb/Aldh2/Aldh7a1 |
| KEGG_PROTEASOME | 7 | 1.77 | 2.10E-02 | 1.11E-01 | 9.23E-02 | Psme2/Psmd8/Psmc3/Psma6/Psmd11/Psmc6 |
| KEGG_GAP_JUNCTION | 13 | 1.73 | 2.46E-02 | 1.27E-01 | 1.05E-01 | Gucy1a1/Tuba4a/Map2k1/Tubb4b/Gnai2/Tuba1b |
| KEGG_ENDOCYTOSIS | 26 | 1.72 | 3.40E-02 | 1.68E-01 | 1.40E-01 | Cdc42/Clta/Flt1/Mdm2/H2-D1/Arf6/Nedd4/Pard3/Ap2b1/Pdcd6ip/F2r/Chmp2b |
| KEGG_PYRUVATE_METABOLISM | 12 | 1.71 | 3.46E-02 | 1.68E-01 | 1.40E-01 | Pkm/Pklr/Ldhb/Glo1/Aldh2/Aldh7a1/Mdh2 |
| KEGG_STEROID_BIOSYNTHESIS | 3 | 1.54 | 4.01E-02 | 1.90E-01 | 1.57E-01 | Dhcr24/Msmo1/Sqle |
| KEGG_ARRHYTHMOGENIC_RIGHT_VENTRICULAR_CARDIOMYOPATHY_ARVC | 12 | 1.64 | 5.04E-02 | 2.26E-01 | 1.88E-01 | Ctnnb1/Actn4/Itgb6/Actb/Actg1/Itgb5/Itga1/Lmna |
| KEGG_ASCORBATE_AND_ALDARATE_METABOLISM | 2 | 1.47 | 5.02E-02 | 2.26E-01 | 1.88E-01 | Aldh2/Aldh7a1 |
| KEGG_DNA_REPLICATION | 3 | -1.47 | 5.48E-02 | 2.40E-01 | 1.99E-01 | Rfc1/Rpa3 |
| KEGG_COLORECTAL_CANCER | 11 | 1.61 | 5.68E-02 | 2.43E-01 | 2.01E-01 | Myc/Map2k1/Ctnnb1/Rhoa/Mapk8 |
| KEGG_PHENYLALANINE_METABOLISM | 6 | 1.6 | 6.05E-02 | 2.46E-01 | 2.04E-01 | Mif/Aldh3b1/Got1/Ddc/Maoa |
| KEGG_GLIOMA | 9 | 1.58 | 5.97E-02 | 2.46E-01 | 2.04E-01 | Cdkn1a/Map2k1/Mdm2/Calm1/Pdgfb/Calm2 |
| KEGG_PATHWAYS_IN_CANCER | 44 | 1.53 | 6.43E-02 | 2.46E-01 | 2.04E-01 | Col4a1/Cdkn1a/Myc/Cdc42/Tpm3/Map2k1/Mdm2/Ctnnb1/Rhoa/Cdh1/Hsp90b1/Mapk8 |
| KEGG_GLYCOSAMINOGLYCAN_BIOSYNTHESIS_HEPARAN_SULFATE | 3 | -1.45 | 6.46E-02 | 2.46E-01 | 2.04E-01 | Ext1/Hs3st3b1 |
| KEGG_UBIQUITIN_MEDIATED_PROTEOLYSIS | 20 | 1.45 | 6.33E-02 | 2.46E-01 | 2.04E-01 | Map3k1/Sae1/Cdc34/Mdm2/Ddb1/Mid1/Nedd4/Ube2m/Anapc11/Ube2c/Cop1/Rbx1/Ube2w |
| KEGG_METABOLISM_OF_XENOBIOTICS_BY_CYTOCHROME_P450 | 15 | 1.58 | 7.19E-02 | 2.47E-01 | 2.05E-01 | Ephx1/Gsta5/Mgst1/Gsta3/Aldh3b1/Mgst3/Gsto1/Cyp1b1/Gstm5/Gstp1 |
| KEGG_RNA_DEGRADATION | 6 | 1.54 | 6.95E-02 | 2.47E-01 | 2.05E-01 | Hspa9/Eno1/Ttc37 |
| KEGG_BETA_ALANINE_METABOLISM | 6 | 1.54 | 7.17E-02 | 2.47E-01 | 2.05E-01 | Hadha/Aldh2/Aldh7a1/Dpys |
| KEGG_ASTHMA | 5 | 1.48 | 7.21E-02 | 2.47E-01 | 2.05E-01 | H2-DMb1/H2-Aa/H2-Ab1 |
| KEGG_WNT_SIGNALING_PATHWAY | 22 | 1.43 | 7.05E-02 | 2.47E-01 | 2.05E-01 | Myc/Ctnnb1/Ppp2ca/Rhoa/Mapk8/Rock2/Ppp2r1b/Ppp2r1a/Cacybp/Ppard/Jun/Rbx1/Tcf7l2/Prkca/Nkd2/Axin2/Smad3/Vangl1/Sfrp2 |
| KEGG_TGF_BETA_SIGNALING_PATHWAY | 15 | 1.55 | 8.07E-02 | 2.48E-01 | 2.06E-01 | Myc/Ppp2ca/Bmp6/Rhoa/Rock2/Ppp2r1b/Ppp2r1a |
| KEGG_FC_GAMMA_R_MEDIATED_PHAGOCYTOSIS | 12 | 1.55 | 8.08E-02 | 2.48E-01 | 2.06E-01 | Cfl1/Cdc42/Arpc5/Map2k1/Arf6/Cfl2 |
| KEGG_FATTY_ACID_METABOLISM | 12 | 1.53 | 8.31E-02 | 2.48E-01 | 2.06E-01 | Eci1/Hadha/Acsl4/Acsl5/Aldh2/Aldh7a1 |
| KEGG_DRUG_METABOLISM_CYTOCHROME_P450 | 15 | 1.52 | 8.52E-02 | 2.48E-01 | 2.06E-01 | Gsta5/Mgst1/Gsta3/Fmo1/Aldh3b1/Mgst3/Gsto1/Maoa/Gstm5/Gstp1 |
| KEGG_NEUROACTIVE_LIGAND_RECEPTOR_INTERACTION | 30 | -1.52 | 7.41E-02 | 2.48E-01 | 2.06E-01 | P2ry13/Npy5r/Drd2/Gabre/Fpr2/Tacr2/Adra2c/P2rx7/Npy4r/Cckar/Lhb/Nr3c1/Chrne/P2rx6/Plg/Mc5r/Chrna6/Crhr2/Taar9/Gabbr1/Prl/Avpr1b/Gnrhr/Gabrr2/Rxfp2/Adrb2/Bdkrb2/Gabrg2 |
| KEGG_ARGININE_AND_PROLINE_METABOLISM | 8 | 1.48 | 8.22E-02 | 2.48E-01 | 2.06E-01 | Ckmt1/Ckb/Aldh2/Aldh7a1/Got1 |
| KEGG_VIRAL_MYOCARDITIS | 11 | 1.48 | 8.41E-02 | 2.48E-01 | 2.06E-01 | H2-DMb1/H2-Aa/H2-D1/Actb/Actg1/H2-Ab1 |
| KEGG_VIBRIO_CHOLERAE_INFECTION | 11 | 1.46 | 8.41E-02 | 2.48E-01 | 2.06E-01 | Atp6ap1/Actb/Actg1/Atp6v1e1/Atp6v0c/Atp6v0e/Atp6v1b2/Prkca/Sec61g/Adcy9 |
| KEGG_FRUCTOSE_AND_MANNOSE_METABOLISM | 4 | 1.45 | 8.10E-02 | 2.48E-01 | 2.06E-01 | Tpi1/Aldoa/Aldob |
| KEGG_ERBB_SIGNALING_PATHWAY | 16 | 1.44 | 8.80E-02 | 2.48E-01 | 2.06E-01 | Cdkn1a/Nrg1/Myc/Map2k1/Btc/Mapk8 |
| KEGG_BASAL_TRANSCRIPTION_FACTORS | 2 | 1.39 | 8.71E-02 | 2.48E-01 | 2.06E-01 | Gtf2h2/Gtf2h1 |
| KEGG_HYPERTROPHIC_CARDIOMYOPATHY_HCM | 13 | 1.45 | 9.38E-02 | 2.60E-01 | 2.16E-01 | Tpm3/Itgb6/Actb/Actg1/Itgb5/Tpm4/Itga1/Lmna |
| KEGG_VASCULAR_SMOOTH_MUSCLE_CONTRACTION | 18 | 1.44 | 9.88E-02 | 2.70E-01 | 2.24E-01 | Ppp1cb/Myl6/Gucy1a1/Map2k1/Rhoa/Rock2/Calm1/Pla2g12a |
| KEGG_ALLOGRAFT_REJECTION | 6 | 1.43 | 1.14E-01 | 2.93E-01 | 2.43E-01 | H2-DMb1/H2-Aa/H2-D1/H2-Ab1 |
| KEGG_TYPE_I_DIABETES_MELLITUS | 6 | 1.43 | 1.14E-01 | 2.93E-01 | 2.43E-01 | H2-DMb1/H2-Aa/H2-D1/H2-Ab1 |
| KEGG_DILATED_CARDIOMYOPATHY | 13 | 1.39 | 1.11E-01 | 2.93E-01 | 2.43E-01 | Tpm3/Itgb6/Actb/Actg1/Itgb5/Tpm4/Itga1/Lmna |
| KEGG_ENDOMETRIAL_CANCER | 9 | 1.38 | 1.15E-01 | 2.93E-01 | 2.43E-01 | Myc/Map2k1/Ctnnb1/Cdh1 |
| KEGG_EPITHELIAL_CELL_SIGNALING_IN_HELICOBACTER_PYLORI_INFECTION | 13 | 1.38 | 1.15E-01 | 2.93E-01 | 2.43E-01 | Cdc42/Mapk8/Atp6ap1/Atp6v1e1/Atp6v0c/Jun/Atp6v0e/Pak1/Atp6v1b2 |
| KEGG_AMINOACYL_TRNA_BIOSYNTHESIS | 6 | 1.41 | 1.21E-01 | 3.00E-01 | 2.49E-01 | Farsa/Aars/Iars/Nars/Sars |
| KEGG_TYPE_II_DIABETES_MELLITUS | 5 | 1.36 | 1.22E-01 | 3.00E-01 | 2.49E-01 | Pkm/Pklr/Mapk8 |
