# Supplementary figures and images for: Compartment-specific adaptive responses and dysregulation under NQO1 deficiency in diabetic kidney disease: A transcriptomic GSEA-based investigation
Source: PLoS One. 2025 Sep 8;20(9):e0331582. doi: 10.1371/journal.pone.0331582 (PMC12416748; doi:10.1371/journal.pone.0331582)

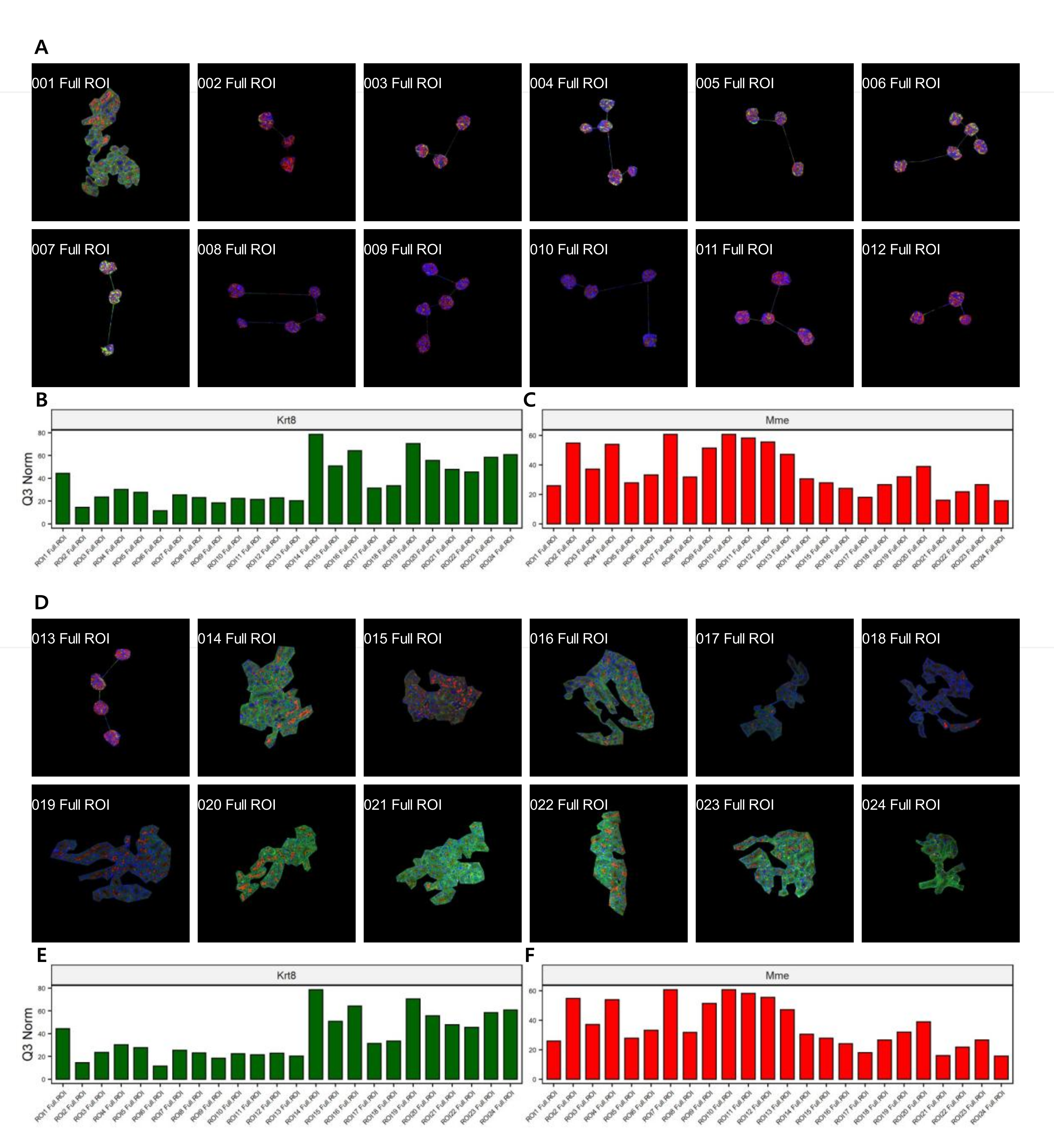

Supplement: S1 Fig — (A, D) Representative kidney sections showing ROIs selected based on tissue morphology, including both injured and intact regions. Staining for CD10, CD31, PanCK, and nuclear dyes guided ROI selection, refined by gene markers (Krt8, Mme, DNA). (B, C, E, F) Quantification of Krt8 (B, E) and Mme (C, F) expression in ROIs, normalized using Q3, illustrating spatial gene expression variation. (TIFF) [file pone.0331582.s004.tiff]
