# Supplementary material for: Compartment-specific adaptive responses and dysregulation under NQO1 deficiency in diabetic kidney disease: A transcriptomic GSEA-based investigation
Source: PLoS One. 2025 Sep 8;20(9):e0331582. doi: 10.1371/journal.pone.0331582 (PMC12416748; doi:10.1371/journal.pone.0331582)
Supplement: S2 File — (PDF) [file pone.0331582.s006.pdf]

【별지 제6호 서식】

동물실험승인서

과제구분	☑ 신규
	□ 계속

(과제코드)<br>과 제 명	( )<br>신장 사구체 손상의 억제에 있어서 자가탐식과 세포골격의 역할 규명
교육(연구)책임자	의과대학 의학과 최대은
승 인 번 호	202112A-CNU-201 (접수번호: 202112-CNU-220)
실 험 기 간	2022년 01월 01일 ~ 2022년 12월 31일

평가 항목에 따른 심의 결과				
1	동물실험 필요근거		☑ 적합	□ 보완
2	동물실험의 대체방법 사용가능성		☑ 확인	□ 미확인
3	동물실험절차 및 동물관리		☑ 적합	□ 보완
4	동물종류 및 계통 선택사유		☑ 적합	□ 보완
5	동물수의 적정성		☑ 적합	□ 보완
6	동물의 인도적 처리기준		☑ 적합	□ 보완
7	실험 중/종료 시 동물의 고통 감소 방안		☑ 적합	□ 보완
8	실험 중 실험동물의 고통과 스트레스 정도		☑ 적합	□ 보완
9	실험물질이 동물과 환경에 미치는 영향		☑ 고려	□ 비고려
10	기타	「동물보호법」 제24조에 해당되는 경우	□ 해당	☑ 해당 없음
		동물실험 실시자 및 관계자의 교육 및 훈련 정도	☑ 적합	□ 보완
위원회<br>의 견	없음			

「충남대학교 동물실험윤리위원회 설치·운영 규정」 제9조제3항과 관련하여 위원회의 심의결과에 따라 위 과제의 동물실험을 승인하였기에 이를 통보합니다.

2021 년 12 월 20 일

충남대학교 동물실험윤리위원회 위원장
